# Martian slope streaks as plausible indicators of transient water activity

**DOI:** 10.1038/s41598-017-07453-9

**Published:** 2017-08-01

**Authors:** Anshuman Bhardwaj, Lydia Sam, F. Javier Martín-Torres, María-Paz Zorzano, Ricardo M. Fonseca

**Affiliations:** 1 Division of Space Technology, Department of Computer Science, Electrical and Space Engineering, Luleå University of Technology, Luleå, Sweden; 2 Institut für Kartographie, Technische Universität Dresden, Dresden, Germany; 3 Department of Environmental Science, Sharda University, Greater Noida, India; 4 Instituto Andaluz de Ciencias de la Tierra (CSIC-UGR), Armilla, Granada Spain; 5 Centro de Astrobiología (INTA-CSIC), 28850 Torrejón de Ardoz, Madrid Spain

## Abstract

Slope streaks have been frequently observed in the equatorial, low thermal inertia and dusty regions of Mars. The reason behind their formation remains unclear with proposed hypotheses for both dry and wet mechanisms. Here, we report an up-to-date distribution and morphometric investigation of Martian slope streaks. We find: (i) a remarkable coexistence of the slope streak distribution with the regions on Mars with high abundances of water-equivalent hydrogen, chlorine, and iron; (ii) favourable thermodynamic conditions for transient deliquescence and brine development in the slope streak regions; (iii) a significant concurrence of slope streak distribution with the regions of enhanced atmospheric water vapour concentration, thus suggestive of a present-day regolith-atmosphere water cycle; and (iv) terrain preferences and flow patterns supporting a wet mechanism for slope streaks. These results suggest a strong local regolith-atmosphere water coupling in the slope streak regions that leads to the formation of these fluidised features. Our conclusions can have profound astrobiological, habitability, environmental, and planetary protection implications.

## Introduction

Exploration of Martian surface features is important to understand its landscape evolution, geochemistry, climatic shifts, and geological regimes^1^. On the other hand, finding evidence of liquid water is a prominent domain of Mars research with implications for the conditions promoting habitability^1–3^ and the future of Mars exploration^2, 4^. The study of Martian landforms not only provides outstanding information regarding past water activity but also, thanks to orbiter high-resolution observations^5^ and data from rovers^3^, we recently had the opportunity to inspect surface features that provide evidence for present-day transient water activity. Slope streaks are one such Martian surface feature, frequently observed in the equatorial low thermal inertia and high dust index regions^6–23^ with debatable implications for both dry^6–10^ and wet^11–16^ mechanisms. Slope streaks have darker albedo than their surroundings^13, 17^ that gradually brightens over decadal timescales causing their fading and appearance as light slope streaks, and finally their disappearance^22^. Several studies have described their characteristics^1, 7, 9–16^ and differentiated them from recurring slope lineae (RSL)^1, 17^. The first observations of slope streaks date back to some of the best resolution Viking Orbiter images^8, 10, 11, 18^. Since then, several researchers have compiled data on their dimensions, patterns, occurrences, and topography^19–23^.

The initial and most widely accepted hypotheses on the formation of slope streaks suggest the involvement of dry processes^6–10^. These hypotheses attribute slope streak formation to a wide range of geophysical phenomena of different scales and natures such as, dark weathered debris^8^, instabilities in a dust mantle by subsurface mobilized debris^18^, air fall deposits and subsequent dust avalanches^6, 9, 10, 23^, enhanced aeolian scars due to photometric effects^24^, dust avalanches on the surface due to the instability caused by subsurface melting^16^, and localized disturbances caused by rockfalls, impact blasts and quakes^7^. In contrast to the dry slope streak models, another body of research proposes models including wet mechanisms behind the slope streak origins. These models attribute the slope streak formations to wet debris flows^11^, transient groundwater springs derived from ground ice^12^, melting frost or ice^16^, seasonal chlorine brines^13^, and low-volume seeps of transient liquids resulting in newly precipitated low-albedo iron oxides^15^. Both dry and wet models are unable to explain several manifestations of slope streaks. In particular, the dry mass wasting or dust avalanching processes cannot explain the following: (i) the undisturbed topography and rock distribution and the absence of debris accumulation at the margins or ends unlike terrestrial rock/sand/snow avalanches and landslides^13^; (ii) the absence of slope streak-like dry granular flow in the terrestrial environment, as the reported analogues from the Antarctic^13, 25^ involve aqueous processes; and (iii) the fact that the streaks are capable of covering kilometer-scale distances on rather gentle slopes (7°–15°)^13, 21^. Due to the lack of visible evidence for significant mass movements in the slope streaks, the inertia and momentum needed to overcome the kinetic angle of repose of 25°–30° for the Martian gravity^26^ is not attainable on these gentle slopes. Moreover, there are also published accounts^23^ against the dry models for regional-scale manifestations such as quakes causing the dry granular flows or slope streaks. On the other hand, some of the intrinsic features of slope streaks remain unexplained through the wet mechanism models. In particular, (i) the groundwater discharge hypothesis faces strong criticism based on surface layer occurrence of the slope streaks and their inconsistency with the bedrock geology^13, 21^; (ii) transient aqueous flow mechanisms are not effectively supported by the observed lack of seasonality in the slope streak formation^23^; (iii) slope streaks can climb over small obstacles of 1–2 m^21^; and (iv) they initiate only over a slope threshold (∼20°), which also argue against the possible involvement of wet processes^21^.

This ongoing debate on one of the most active surface manifestations on present-day Mars receives a new perspective through our study. We realized the need for a multi-scale study focusing on both a high-resolution survey and inventory of the distribution of these features and a high-resolution local terrain morphometric analysis to understand their topographic preferences. Here, we report an up-to-date distribution and morphometric investigation of the Martian slope streaks. We further correlate the distribution map of slope streaks with various physio-chemical parameters of the Martian regolith and atmosphere to characterise the slope streaks and understand their origin.

## Results and Discussion

### Global distribution and chemical concurrences

**Figure 1:**
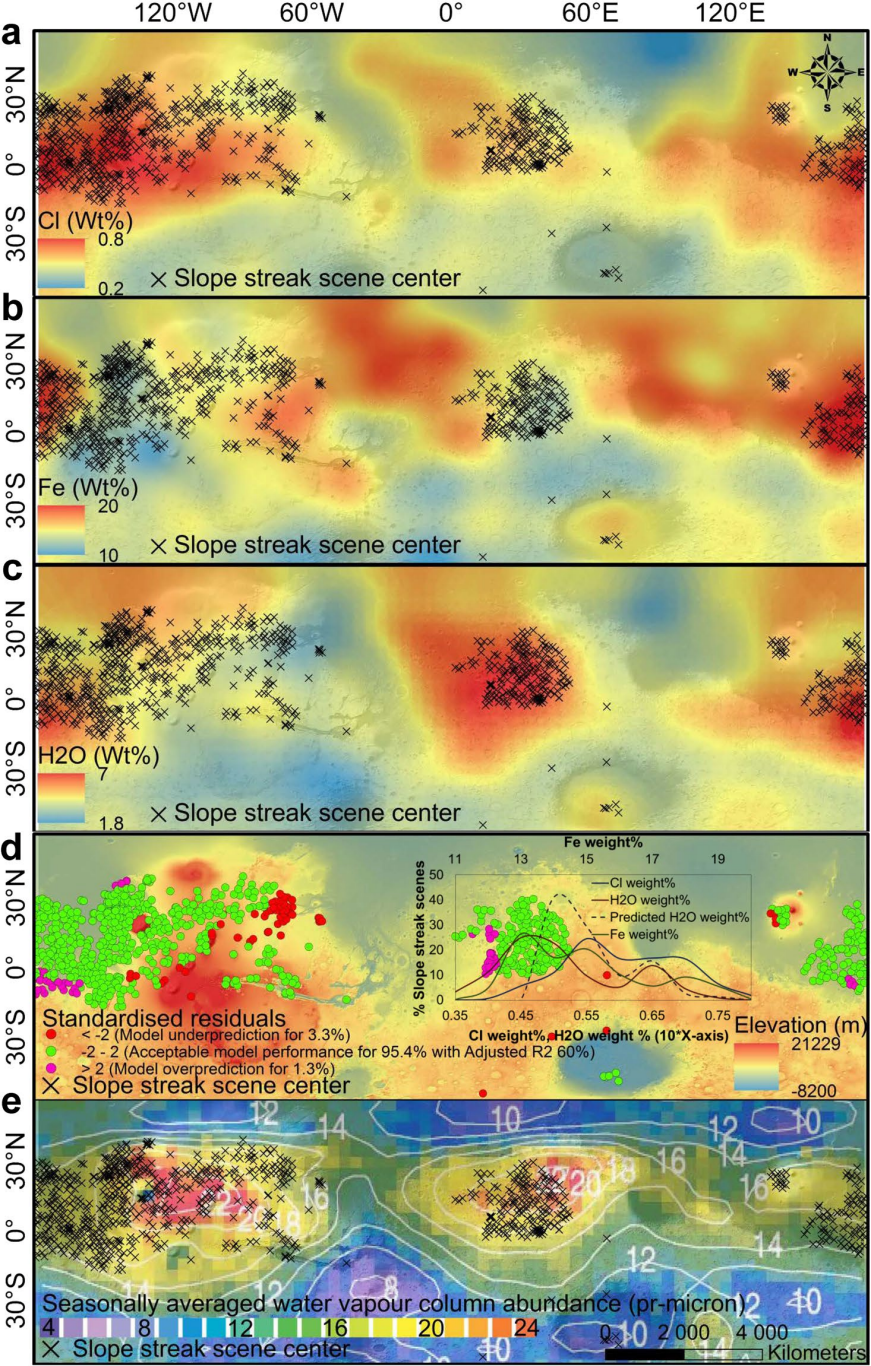
Global distribution of slope streaks and chemical parameters. (**a**) Cl concentration^30, 31^. (**b**) Fe concentration^31, 32^. (**c**) Water-equivalent hydrogen concentration^30, 31^. (**d**) Geographically weighted regression at the 95% confidence level^33^. The inset graph shows the % of SSR scenes in various concentration categories. (**e**), Mars Global Surveyor Thermal Emission Spectrometer (TES)-derived seasonally averaged water vapour column abundance after negating the effect of topography (modified Fig. 10 of Smith^34^). The numbers and contours represent seasonally averaged water vapour column abundance in e. All of the weight% values given in a-c are taken from https://grs.lpl.arizona.edu/grs-web/specials/Smoothed_rebinned_map_data. Mars Orbiter Laser Altimeter (MOLA) elevation-derived hillshaded view is in the background of a-e. The Maps are created using ArcGIS Version 10.4 (http://desktop.arcgis.com/en/arcmap/latest/get-started/setup/arcgis-desktop-quick-start-guide.htm).

During the past decade, nearly the entire Martian terrain has been captured at an unprecedented high spatial resolution using the Mars Reconnaissance Orbiter (MRO) ConTeXt (CTX) imager and the High Resolution Imaging Science Experiment (HiRISE) camera^27^. We performed a systematic survey of all of the available images (detailed in the Materials and Methods section) to derive a distribution map of slope streaks on Mars. Several studies in the past have tried to survey the global distribution of slope streaks^19, 22^ using image samples. Here, we cover ~95% of the region where slope streaks appear, i.e., Martian terrain between ±60° latitudes, at high resolutions to confirm the global distribution of slope streaks (Supplementary Fig. 1a). A geo-statistical optimised hot spot analysis^28, 29^ highlights three of the hot spots within 99% confidence limits (Supplementary Fig. 1c): (i) west of Olympus Mons, (ii) the southern part of Arabia Terra, and (iii) south of Elysium Rise. We plot the observed slope streak sites on the global maps of Martian regolith physio-chemical parameters (Fig. 1 and Supplementary Fig. 2). Our high-resolution analysis of the slope streaks of the Elysium Planitia region shows a higher appearance rate (12% new streaks per existing streak per Martian year) than previously documented^19, 20, 22, 23^ (described in Materials and Methods). Additionally, a systematic analysis of high-resolution images helps us to locate several Slope Streak Regions (SSR) in western Kasei Valles, Valles Marineris, and north of Apollinaria Patera. These new findings highlight the global-scale distribution and frequent occurrences of the slope streaks.

The fact that the slope streaks do not display any seasonality^11–16^ or lengthening suggests that the probable wet phase of these features is short-lived and they are formed suddenly due to the transient attainment of a delicate balance of favourable deliquescence conditions irrespective of the season. Their coexistence with the low thermal inertia regions^6–23^ supports the fact that the SSR display high diurnal variability in surface temperatures and thus have a higher possibility of ephemeral water scenarios. If slope streaks do not show seasonality, they appear and grow in short-lived processes that can happen at any plausible time of the year. The diurnal variation of the surface temperatures (T) on Mars can be extremely large (typically reaching up to 90 K)^3^ and thus the variation of Relative Humidity (RH) over one single day is larger than that over the full year giving a large range of plausible states over diurnal T/RH phase-diagram. Thus a transient balance of deliquescence conditions may be sufficient to launch the formation of slope streaks. We start with the hypothesis that a diurnal regolith-atmosphere water coupling in the SSR governs the formation mechanism of slope streaks.The main requirements for a plausible wet mechanism behind the origin and distribution of the slope streaks are as follows: (i) the presence of eutectic salts (hydrated or anhydrous) in the regolith; and (ii) suitable temperature and RH conditions for salt eutectics. Chlorine (Cl) is the most vital chemical component of the Martian regolith that is capable of forming various chlorides and perchlorate salts with extremely low eutectic temperatures and high solubilities^5^. Figure 1 shows the positive associations between Cl^30, 31^, Fe^31^, water-equivalent hydrogen, and SSR, based on data from the Gamma Ray Spectrometer (GRS) aboard the 2001 Mars Odyssey (https://grs.lpl.arizona.edu/grs-web/specials/Smoothed_rebinned_map_data). In order to avoid any confusion, here we clarify that the seemingly lower Fe concentrations (~10%) in case of few of the SSR in Fig. 1b should not be considered as the limiting concentrations for brine formations as they are still in the upper-half concentration range of the global Fe distribution as also observed through the visible near-infrared imaging spectrometer Observatoire pour la Minéralogie, l’Eau,les Glaces et l’Activité (OMEGA) on board the ESA Mars Express (MEx)^32^.The spatial correlations of Cl, regolith hydration levels, and atmospheric water content with the distribution of the SSR suggest that these may be caused by a regolith-atmospheric water interchange mediated by chlorine salts. We performed an advanced geostatistical analysis (detailed in the Materials and Methods section) to quantify the correlation between these parameters (Fig. 1d). Of the total SSR scenes, ~83% display above-average concentrations of Cl (>0.5% by weight), ~82% show the highest regolith hydration levels outside the poles (>4% by weight), and ~95% exhibit the highest Fe concentrations (>12% by weight) (Fig. 1d). The hydration levels (Fig. 1c) in the SSR point towards both a possible association with the salts and possible briny conditions. Taking hydration levels as the dependent variable and Cl (Fig. 1a) and Fe (Fig. 1b) concentrations in the SSR as explanatory or independent variables in the geographically weighted regression (GWR)^33^ analysis, we observe a significantly acceptable model performance at the 95% confidence level. A reclassified map based on standardised residuals (Fig. 1d) of GWR presents a clear picture of collocation among Cl, Fe, water-equivalent hydrogen, and SSR. In the case of 95.4% of the SSR scenes, we observe an acceptable model performance for predicting hydration levels depending on Cl and Fe concentrations with an adjusted R^2^ value of 60%. Only for 4.6% of the scenes is there a significant over- or underprediction. As is clear from the inset graph in Fig. 1d, these over- or underpredictions are prominent in cases of extremely low concentration values of the variables, which can be further attributed to the coarse resolution of the gridded GRS data and data smoothing operations. For the majority of the SSR that display above average variable concentrations, the considerable GWR results show a geographical correlation among the variables. A remarkable observation based on the Mars Global Surveyor Thermal Emission Spectrometer (TES) data for estimating seasonally averaged water vapour column abundance (Fig. 10 of Smith)^34^ indicates that ~95% of SSR coincide with higher than average to the highest water vapour column abundances in equatorial and subequatorial regions (Fig. 1e). Although, the global map of height-corrected water vapour column abundance (Fig. 1e) shows a variability between 4 and 24 pr-µm, ~95% of SSR are located in the regions where the abundance values are above 14 pr-µm and for the SSR hotspots (Supplementary Fig. 1c and e) the contour plots of water vapour column localisation is furthermore very similar to the water distribution (Fig. 1c). Local orography, slope aspects, and topography may favour some local, small scale anisotropies in seasonally averaged water vapour column values, but at the scale of the map averages these features should be negligible as each bin of the map in Fig. 1e is too coarse to comment on the local scale topographic controls (2° wide in latitude and 2° wide in season)^34^. The global circulation effect has been claimed as the reason^34^ for the high water vapour column in these latitudes irrespective of the seasonal polar cap dynamics. However, this does not explain the positional (longitudinal-latitudinal) dependencies of the water vapour column, even when the effect of topography is negated (Fig. 1e; Fig. 10 of Smith^34^). In absence of the topographic influence, the global circulation should not favor one longitude over others to such an extent unless there is a local source and sink of water available. A notable point here is that the deliquescence and efflorescence, or even normal hydration/dehydration, does not significantly depend on H_2_O partial pressure but on RH, and thus, depends critically on temperature (T). Therefore, in the case of a homogenous H_2_O Volume Mixing Ratio (VMR) in the atmosphere, the RH would be driven by latitude and elevation (because these are conditioning T). The way to disentangle the local variations in water vapour column and to find local differences (for a given latitude) is to use the elevation corrected averaged water vapour column abundance map. This high spatial correlation (Fig. 1e) is a plausible indicator of the existence of a present-day regolith-atmosphere water vapour cycle in the SSR mediated by mechanisms that cause the appearance and disappearance of the slope streak features. The salt-hydration-based deliquescence needs moisture from the regolith and/or the atmosphere to form the slope streak features, and the proposed transient nature of these features requires a quick desiccation mechanism involving evaporation or sublimation, thus hinting towards a strong local regolith-atmosphere water coupling. This TES-based water vapour column abundance in the SSR strongly supports our hypothesis of the presence of widespread ephemeral water scenarios in the SSR.

**Figure 2:**
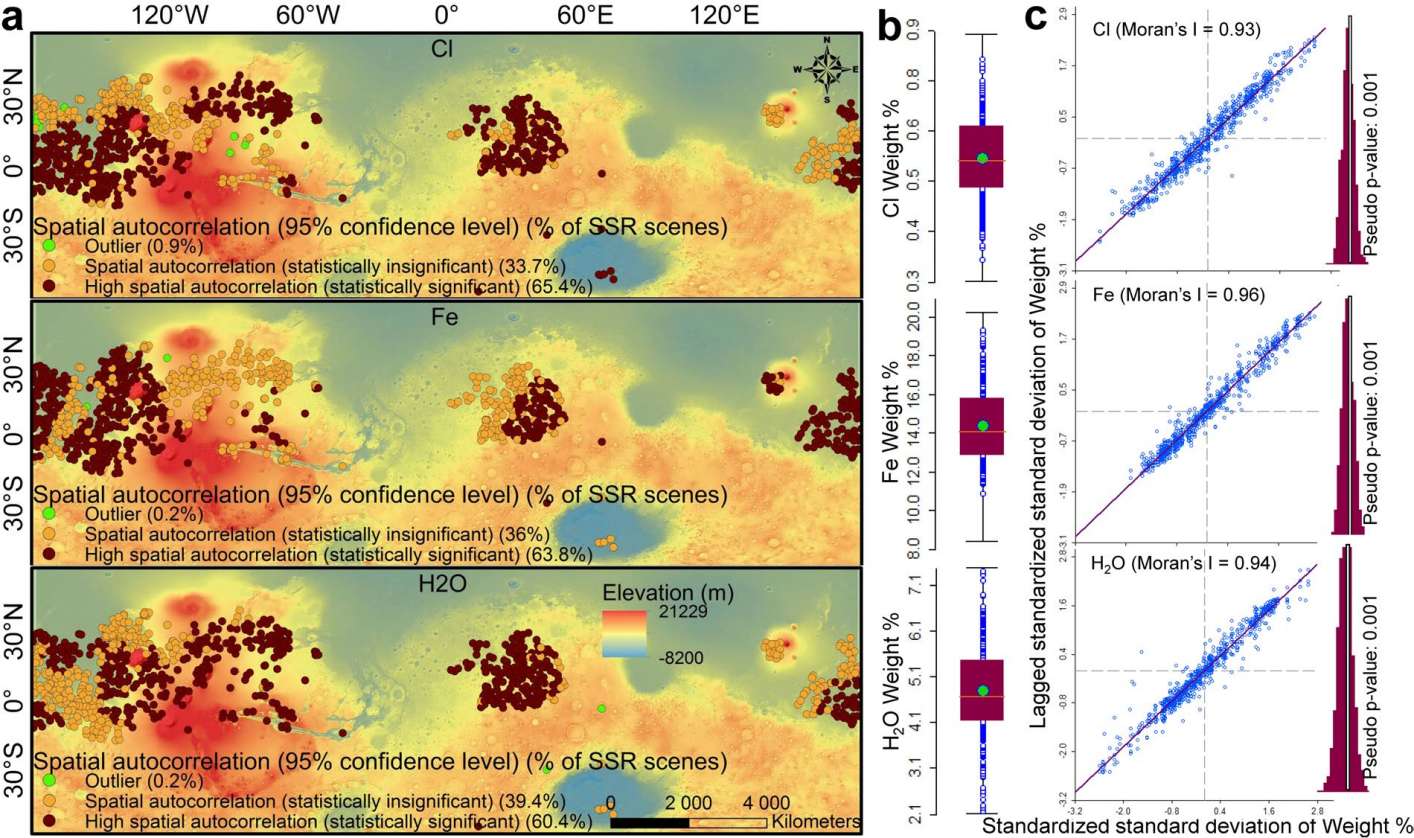
Anselin Local Moran’s I spatial autocorrelations within the geochemical variables in the SSR. (**a**) Spatial autocorrelation at the 95% confidence level. (**b**) Data with no outliers, i.e., normal distribution. The green circle is the average value, the maroon area is the 25% to 75% range of values, the orange line is the median, and the black lines are considered to be the limits for a normal distribution. Any circles outside the black lines are the outliers. (**c**) Moran Scatter Plots^35^. The maroon histograms represent a normal distribution after Moran’s I randomisation at 999 permutations. Mars Orbiter Laser Altimeter (MOLA) elevation-derived hillshaded view is in the background of the maps in a. The Maps are created using ArcGIS Version 10.4 (http://desktop.arcgis.com/en/arcmap/latest/get-started/setup/arcgis-desktop-quick-start-guide.htm).

As an additional step to finding the possible statistically robust spatial autocorrelations within the variables mentioned above, we performed an Anselin Local Moran’s I spatial autocorrelation analysis^35^ (detailed in the Materials﻿ and Methods) and identified the clustering events (Fig. 2). This analysis is to prove that there is no geographical randomness in the high concentrations of Cl, Fe, and H_2_O in the SSR and they certainly display strong spatial clustering. As is evident in Fig. 2b, for the SSR, all the three concentrations (Cl, Fe, and hydration levels) show perfectly normal distributions without presence of any outlier. The maps in Fig. 2a correspond to the spatial autocorrelation for SSR, i.e, the percent of scenes showing similar concentration values as their neighbors’ at the 95% confidence level. In all three cases (Fig. 2a), >60% of the SSR scenes show high spatial correlations, i.e., for >60% of SSR, nearby SSR show similar types of Cl, Fe, and water-equivalent H concentrations in the regolith. This percentage increases to ~90% if we decrease the confidence limit to 90%. The extremely high Moran’s I values of ≥0.93 in the Moran Scatter Plots (Fig. 2c) indicate particularly high autocorrelations, along with statistically significant pseudo p-values of 0.001 and a normal distribution of z-values (histograms in Fig. 2c)^35^, which are sufficient to reject the null hypothesis of geographical randomness (no spatial clustering) in concentration values. Thus, this analysis proves that the SSR not only show the physical similarities of high dust indices and low thermal inertia, but they also prominently display congruence in the concentrations of the chemical species that are necessary to promote ephemeral water scenarios in the Martian regolith. Considering the coarse resolutions of the chemical maps, we certainly imply that >60% of autocorrelation at 95% confidence level and ~90% autocorrelation at 90% confidence level are extremely strong figures to ignore or disqualify our inferences.

### Probable salts and temperature thresholds for salt eutectics

Cl is the most vital chemical component of the Martian regolith that is capable of forming various chlorides and perchlorate salts with extremely low eutectic temperatures and high solubilities^5^. As estimated in the previous section, we have compelling evidence of the concurrence of the SSR with high Cl and Fe concentrations along with high hydration levels. In Fig. 1, Cl^−^ and Fe^3+^ appear to be the key anionic and cationic species, respectively, within the SSR. Slope streak sites south of Syrtis Major and north and west of Hellas Planitia, which are presented here for the first time, coincide with high-calcium pyroxene regions (Supplementary Fig. 2a), suggesting Ca^2+^ as another cationic candidate. Another relevant study^36^ that used GRS data and statistical analysis also places all of our SSR within 3, 4, and 5 of their geochemical provinces, which depict average or greater than average concentrations of Fe and Ca (Fig. 1A of Taylor *et al*.^36^). Additionally, several of the SSR showing comparatively lesser abundance of Fe (e.g., in Arabia Terra and Valles Marineris) in Fig. 1b are expected to be richer in Mg as Mg varies inversely with Fe in the low- and mid-latitudes^31, 32^. Thus, chlorides or perchlorate salts of Fe, Ca, and Mg appear to be the best candidates as freezing point depressants in the SSR. Although recent research has highlighted contact electrification of regolith particles and Cl electrolysis for extensive natural perchlorate synthesis^37^, due to several uncertainties related to such a wide-scale perchlorate distribution^38^, here we consider FeCl_3_, CaCl_2_, and MgCl_2_ as the prevalent salts that are capable of forming eutectic brines in the SSR at a global scale. However, we are not ruling out the possibility of the presence of cationic species such as Na^+^, and K^+^ or the formation of sulfates, nitrates, and carbonates at local scales. The presence of MgSO_4_ and its various hydration states in the equatorial regions of Mars and their linkage with the water cycle have been documented^39^. However, in the case of SSR, the needed freezing point depression for probable ephemeral water scenarios is high (~60 K) and the sulfates cannot achieve such a degree of eutectics^40^. In fact, the eutectic temperature considerations for these salts reveal that FeCl_3_ and CaCl_2_ are the strongest freezing point depressants with brine eutectics at approximately 218 K, followed by MgCl_2_, NaCl, and KCl at approximately 238 K, 252 K, and 262 K, respectively^40^. None of the probable sulfates show a eutectic temperature below 263 K, while the carbonates are either poorly soluble (Ca, Mg) or ineffective as a depressant (Na)^40^. Among the nitrates, the most effective depressant is Mg(NO_3_)_2_, which can bring the freezing point down to 244 K, but their prevalence in the SSR seems to be low.

Our hypothesis is that the wet phase of the slope streaks is extremely short-lived and they are formed suddenly due to transient attainment of a delicate balance of favourable deliquescence conditions independent of seasonality but governed by the diurnal regolith-atmosphere water coupling. The insignificant hydration levels observed for several slope streaks using spectra from the Compact Reconnaissance Imaging Spectrometer for Mars (CRISM) instrument^15^ also indicate that these features are only transiently hydrated. The high possibility of the involvement of FeCl_3_ and CaCl_2_, or a mixture of the ionic species discussed above (particularly MgCl_2_ due to its high eutectics), further supports our hypothesis as FeCl_3_-, CaCl_2_-, and MgCl_2_-containing brines can attract the lithospheric sulfates via acid displacement reactions^40^ to react and solidify the solution, making amorphous precipitates^39^ and forming sulfates and chlorides, which are not equally effective freezing point depressents^40^. Clark and Hart^40^ have described the possibility of such large scale transformations starting with the fact regarding high solubility of all chloride salts of the major cationic species on Mars (Ca, Fe, Na, Mg). FeCl_3_ solutions are strong acids and thermodynamically unstable in the presence of the sulfates prevalent on Mars, readily forming the precipitates and sulfates. These precipitates and byproducts are known to resist complete dehydration for a prolonged duration and even their desiccation does not lead to textural disintegration^39^. Thus, such low-albedo precipitates explain the dark tone and solidified saturated texture of the slope streaks^15^. Additionally, the known difficulty of attaining the required RH and vapour pressure thresholds in the Martian atmosphere suggests the extremely ephemeral wet nature of the slope streaks. Several recent laboratory simulations prove that under the present Martian RH conditions, CaCl_2_^41^ and FeCl_3_^42^ are the chloride salts that can display a level of deliquescence similar to those of the perchlorates. In the case of dihydrate CaCl_2_, the deliquescence relative humidity (DRH) can be as low as 15.8 ± 3.5%, showing a decrease with decreasing hydration state (from the initial hexahydrate state) and with increasing temperature^41^. Similarly, FeCl_3_ shows an initial liquid phase at 11% RH with a subsequent gel-like appearance, after its association with sulfates^42^. In fact, the importance of widely underestimated ferric ion chemistry^43^ in deciding the overall Martian hydro-lithochemistry is gaining acceptance with such scientific reporting.

After a discussion on the probable salts and eutectic temperature thresholds, we consider the surface temperature conditions in the SSR. In view of the prevalence of FeCl_3_ and CaCl_2_ in the SSR, the minimum reachable eutectic threshold for brine development is 218 K^40^. The yearly average surface temperature map of Mars (Supplementary Fig. 3a)^44, 45^ shows a temperature of ≥210 K for ~95% of the slope streak sites falling within 30°N to 50°S latitudes. A careful consideration of RH and diurnal temperature variations in simulated Martian conditions predicts the possibility of metastable brines between 9:00 Local True Solar Time (LTST) and midnight LTST with varying combinations of RH and air temperature starting from 27% and 223 K, respectively (Supplementary Fig. 3b)^41^. Since our hypothesis and discussion above suggest an extremely transient nature for the deliquescence forming the slope streaks, we assume that the maximum diurnal surface temperatures, which have less dependency on the RH, are responsible for the initiation of this deliquescence. Thus, we study the correlation between the average annual maximum surface temperature (Fig. 2b) with the SSR, and as expected, all of the SSR (including the southernmost) display annual maximum surface temperatures ≥223 K (Supplementary Fig. 4). Moreover, the Mars Climate Database (MCD)^44, 45^ also suggests that in all the slope streak hot spots, the maximum surface temperature easily crosses the eutectic freezing point of water at least once every Martian sol (mostly in the afternoon to late evening) throughout the year, thus permitting the possibility of extensive deliquescence in the presence of chloride salts in the regolith. Supplementary Fig. 3b shows the needed temperature threshold at 16:00 LTST to be 242 K for 11% RH, which is easily crossed by the diurnal surface temperatures in the SSR. The relevant entity for deliquescence is not the amount of water but the RH%; salts will absorb moisture from the atmosphere to hydrate and then deliquesce. The RH reaches high values (even saturation) as soon as the temperatures on Mars starts dropping on a diurnal basis; even at the equatorial latitudes such as the Gale crater the diurnal RH reaches saturation levels at the surface^3^. All the calculations of atmospheric water assume that water vapour is uniformly mixed in the vertical height within the boundary layer, but this is certainly not the case if there is a diurnal interchange of water. This discussion has further been elaborated in the Supplementary information. However, the fact remains that when the RH is so high, instantaneous deliquescence can occur even on the smallest grain size.

As noted above, the coexistence of the SSR with the low thermal inertia regions implies high diurnal variations in the surface temperatures and thus a higher possibility of evaporation or rapid freezing of the formed brines and their byproducts (usually sulfates). If freezing occurs, it further keeps the dusty regolith covered by slope streaks hyper-saturated and dark-toned, and the hydration levels cannot be detected by optical hyperspectral sensors^15^ after deposition of even a thin film of dust subsequent to any episode of the frequent dust storms in these regions. Once frozen, consecutive melting within such slope streaks becomes extremely difficult due to changed hydration levels and salt contents of the regolith. This is the reason why slope streaks regenerating from the same point and covering the same region or showing some elongation have rarely been reported. However, we for the first time report several examples of such slope streaks in Supplementary Fig. 5a and b, which can regenerate only after a sufficient time lag, probably due to their separate desiccation mechanism (evaporation and not freezing), or the deposition of some dust and associated salts and attainment of the needed RH and temperature conditions (in the case of freezing). However, an interesting aspect is that despite the year-round high solar insolation (Supplementary Fig. 6a) and high diurnal maximum surface temperature variability in these regions, the average sol-to-sol maximum surface temperature variability is <2 K (Supplementary Fig. 6b). This fact combined with the high water vapour column throughout the year (Fig. 1e) further explains the SSR’s non-dependence on the seasons and suggests that freezing of the brines is not the only possibility but that there are even higher chances of them drying up through rapid evaporation or sublimation. Given their latitudinal placement, we propose sublimation as the active process involved in desiccation and fading of the southernmost slope streaks observed in Russell Crater (Supplementary Fig. 7)^46^. The morphological difference between the sublimated arid slope streaks and the CO_2_ sublimation gullies (Supplementary Fig. 7b) signifies the involvement of H_2_O in the former case (detailed in the Supplementary information). The plausible water activity may be further confirmed through the slope orientation of all the southernmost slope streaks, i.e., northwesterly or facing the afternoon sun (Supplementary Fig. 7), thus representing the optimal time of the day for brine formation (Supplementary Fig. 3b)^41^.

### Terrain preferences and flow patterns

There are three major constraints reported in the literature against the wet mechanisms for the slope streaks: (i) lack of seasonality^23^; (ii) their possible initiation only over a slope threshold (∼20°)^21^; and (iii) the ability of the slope streaks to climb over small obstacles^21^. In the previous section, we present clear evidence to highlight the non-dependence of the plausible wet scenarios on a particular season and their dependence on a diurnal cycle. In this section, we eliminate the other two constraints through a careful consideration of the terrain preferences and flow patterns displayed by the slope streaks.

**Figure 3:**
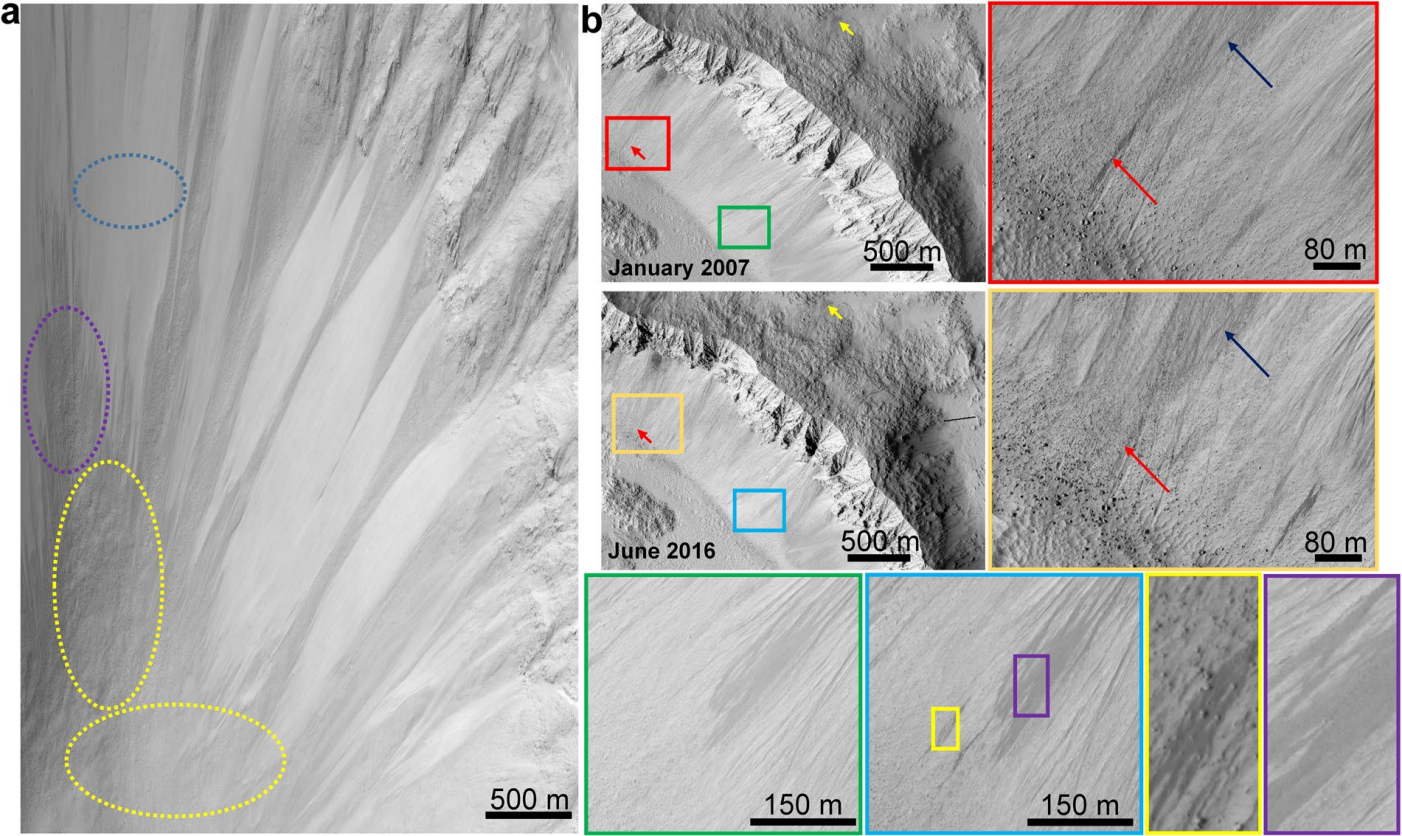
Difference between low-volume mass wasting and slope streaks in HiRISE images. (**a**) Scree falls and talus deposits. The blue dotted ellipse differentiates the smooth texture of these mass movements from the rough-textured bed rock (violet dotted ellipse). The yellow dotted ellipse shows the hazy texture corresponding to the downslope dust and talus deposits. (**b**) Streaks and mass wasting in Zunil Crater. The smaller red and yellow arrows in the upper left panel (January 2007) show similar albedo streaks on steep and gentle slopes, respectively. The orange rectangle in the June 2016 image shows the disappearance of the steep slope streak (in red rectangle). The blue arrows show the active mass movement slopes above the slope streak. The green and blue rectangles highlight the appearance of a new streak (marked by yellow rectangle). The yellow and violet rectangles show the difference in topography of the slope streak and the low-volume mass wasting, respectively, on the same slope. HiRISE image credit: NASA/JPL/University of Arizona.

First, we maintain that contrary to the prevalent assumption^6–10^, the slope streaks are definitely not mass movements. A detailed literature survey suggests that the possible mass movements morphologically and volumetrically closest to the slope streaks can be either dust avalanches or scree falls. Supplementary Fig. 5c shows several slope streaks with a flow pattern typical of fluids, i.e., the branching at an obstacle followed by a convergence due to the cohesive forces and covering long distances on nearly flat slopes due to the capillary support and high adsorption provided by the dusty arid regolith. Figure 3 noticeably depicts the topographical and textural differences between low-volume mass movements such as scree falls and the slope streaks. In Fig. 3a, the blue dotted ellipse shows the smooth texture of these mass movements, which is significantly different from the rough-textured bedrock (violet dotted ellipse). It certainly appears to be a surface phenomenon that covers the underlying bedrock. Moreover, the yellow dotted ellipses (Fig. 3a) show the hazy texture corresponding to downslope dust and talus deposits. These appearances are not characteristic of the slope streaks. To further characterise these differences, we present an example from Zunil Crater in Fig. 3b. This crater is the best example for observing mass movements and slope streaks on the same slopes. We selected Zunil Crater for this discussion because it is well-known for several recent and persistent dry mass movements (e.g., http://hirise.lpl.arizona.edu/PSP_001764_1880, Supplementary Fig. 8). Our hypothesis presented in the paper is against any kind of mass movement, whether dry or wet, since we (and several past studies)^12, 13, 21, 25^ observe the undisturbed topography and the absence of debris deposits at the base of the slope streaks (Fig. 3), acknowledge the dry mass movement propagation constraints in the Martian gravity^13, 21^, and conform to the fact of absence of the dry mass movements in any reported terrestrial analogue^13, 21, 25^. A study by Phillips *et al*.^9^ displays the appearance of several mounds within a slope streak and suggests probable sediment movement. However, such observations are inconsistent at a wider scale and cannot be generalised. Nevertheless, based on the volumetric estimates, Phillips *et al*.^9^ make a valid point that dust may be transported without surface albedo change and thus necessarily associating mass movements with albedo changes can be misleading while interpreting the Martian surface features. In addition, Phillips *et al*.^9^ also mention that mass movement or sediment transport does not eliminate the possibility of an aqueous process as they find the measured slope of the studied slope streak to be lesser than the angle of repose, thus supporting the wet models^11, 12, 25^. Here, we are focusing our discussion on plausible brine propagation through the dusty regolith for an extremely transient duration. We agree that such brine propagation has the capability to initiate further mass movements on the surrounding slopes or downslopes, but the truth remains that such mass movements associated with slope streaks have rarely been shown or discussed. While mentioning all these arguments, we still acknowledge the option of granular flow within the slope streaks as we understand the spatial resolution limitations of the remote sensing data. Our observations of the undisturbed topography of the slope streaks are based on the sub-meter resolution HiRISE images. Such resolution is although good to observe the mass movements; we understand the possibility of the granular flows which can be of an even finer resolution difficult to be observed through the HiRISE images. Based on viscoplastic flow modeling and photoclinometry, Miyamoto *et al*.^14^ also suggest probable fluid rheology (water-related flow with <20% solid content) and a short formation period for the slope streaks. Additionally, they rule out the possibility of any debris flow in the slope streaks and suggest the possibility of any probable microscopic dry grain flow only through the involvement of dispersive pressure or a lubricant (possible fluidized mechanism). The mass movements depicted in Fig. 3 for comparing with the slope streaks are dry or wet, that can be another topic of discussion. However, here we will consider them to be dry by establishing an analogy with the confirmed lunar dry mass movements detected using Lunar Reconnaissance Orbiter Camera (LROC) Narrow Angle Cameras (NACs) (Supplementary Fig. 9). In fact, not just on lunar slopes but such dry mass movements are also prevalent even on the loose talus slopes of terrestrial high-mountains such as the Himalayan ranges (Supplementary Fig. 9). Here, we additionally intend to mention the morphological analogues on Earth displaying similar fluidised flow as the slope streaks, and contradicting the dry mass movement hypotheses. Such analogues have been reported from the McMurdo Dry Valleys in Antarctic by several researchers^25^ (Supplementary Fig. 10) where they have displayed the absence of dry granular flow and confirmed the reported analogues to be consisting of fluidised flow. In Fig. 3b, the smaller red and yellow arrows in the upper left panel (January 2007) show similar albedo streaks on steep and gentle slopes, respectively, implying a similar time of initiation for them. However, the orange rectangle in the image from June 2016 shows the complete disappearance of the steep slope streak (in red rectangle), while the gentle slope streak is still clearly visible with little evidence of fading. The blue arrows in the June 2016 image show the active mass movement slopes above the steep slope streak that explain its complete fading due to dust accumulation. The green and blue rectangles highlight the appearance of a new streak (marked by yellow rectangle). The yellow and violet rectangles show the difference in topography of the slope streak and the low-volume mass wasting, respectively, on the same slope. The rough-textured bedrock is visible in the yellow rectangle, while the smooth dusty deposit covering the underlying bedrock in the violet rectangle clearly differentiates between the two processes.

**Figure 4:**
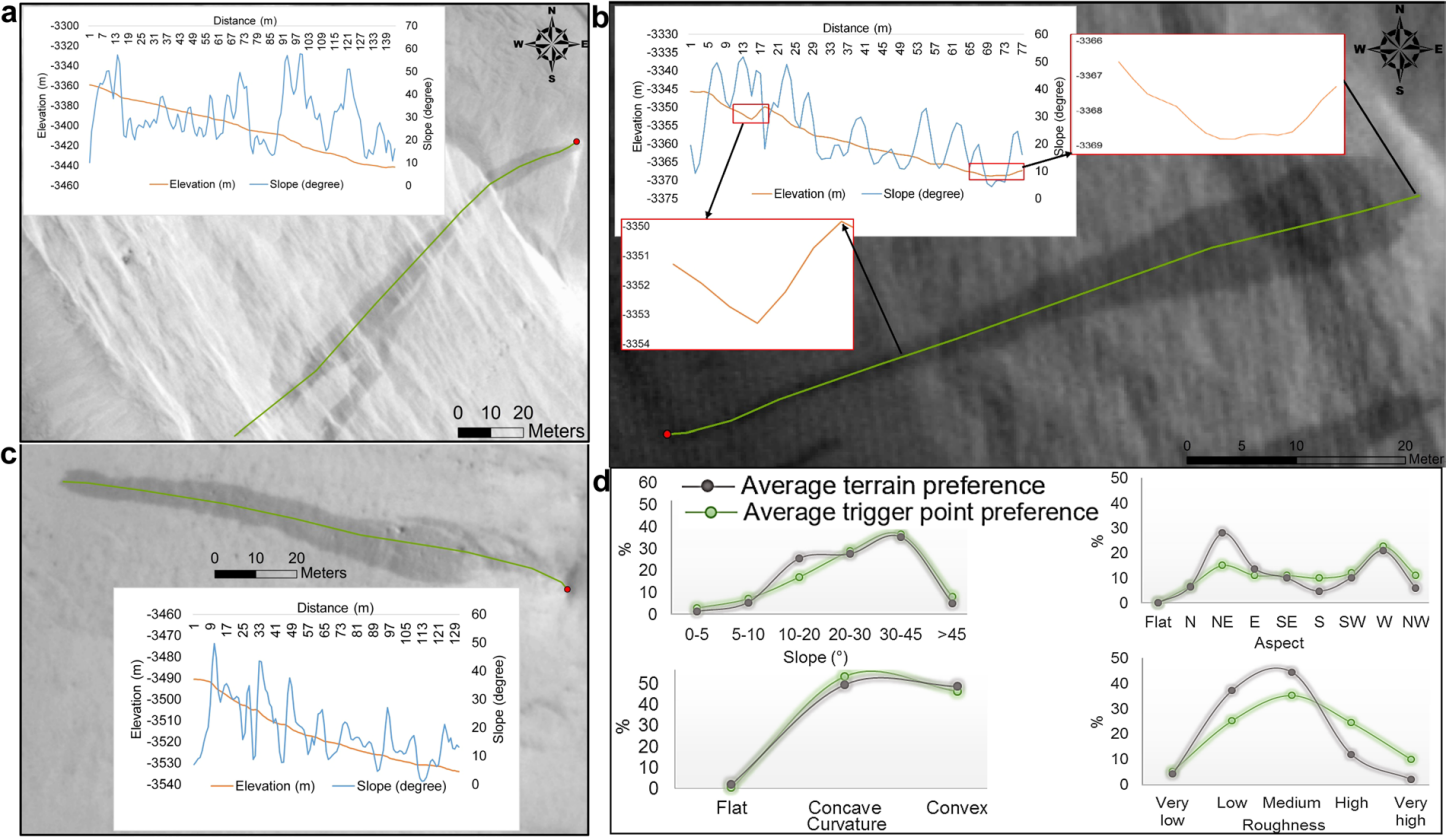
Terrain preferences and slope characteristics revealed by HiRISE images and DTMs. (**a**,**b** and **c**) Slope streaks over widely variable slopes. Red dots represent the point of initiation and green curves are used for the transect analysis of the terrain. The highlighted plots within red rectangles in Fig. 4b show the slope streak movement against gravity. (**d**) Geomorphometric preferences displayed by the slope streaks. The Maps are created using ArcGIS Version 10.4 (http://desktop.arcgis.com/en/arcmap/latest/get-started/setup/arcgis-desktop-quick-start-guide.htm). HiRISE image credit: NASA/JPL/University of Arizona.

In the next step, we perform a high-resolution morphometric analysis using several HiRISE Digital Terrain Models (DTMs) covering the SSR between the equator and 30°N latitude (Supplementary Fig. 1b) (detailed in the Materials and Methods). To offer a statistical robustness and an unbiased scenario to the interpretations, we include ~1,000 slope streak samples within this analysis. The length, maximum width, and area vary from 14 m, 1 m, and 6 m^2^, respectively, for a streak in Arabia Terra to 1,600 m, 148 m, and 84,800 m^2^, respectively, for a streak in Tooting Crater. Figure 4a,b and c depict the downslope movement of slope streaks over widely variable slopes. Similar to Fig. 4a and c, we can find numerous examples (~8%, Fig. 4d) where the slope streak initiation occurs on nearly flat to gentle slopes (<10°), significantly lesser than the earlier reported slope threshold (∼20°)^21^ and thus, not supportive of dry mass movements^26^. We believe that the process of deliquescence starts as a point phenomenon on the curved and rugged slopes (Fig. 4d) as they ensure fewer dust deposits than on the flat terrain. This relatively thinner dust layer is thus more vulnerable to exposing the underlying mineralogy to the deliquescence conditions of solar fluxes, temperatures, and humidity due to any factor strong enough to remove this moderate dust layer (rock fall impact or strong wind). In fact, the aspect distribution in Fig. 4d also shows that ~70% of the slope streaks initiate on slope orientations following the diurnal movement of the Sun (east-northeast to west-southwest), and thus, the streaks are more of a diurnal phenomenon than a seasonal one. Supplementary Fig. 6a further represents the SSR to have the highest average annual solar radiative flux to surface. Once the deliquescence starts due to the hydrated salt eutectics and moisture in the regolith or the atmosphere, depending on the duration of the transient balance between the required conditions, the brine can cover a wide range of distances, elevations, and slopes taking advantage of the capillary support and high adsorption provided by the dusty arid regolith of the SSR. Moreover, the high adsorption and capillarity coupled with the low gravitational pull of Mars are the reasons behind the ability of the slope streaks to climb obstacles as high as 3.5 m (Fig. 4b), contrary to the previous estimates of 1–2 m^21^. Water movement within the soil is governed by the water potential difference, gravity, capillary action, and soil porosity. In the case of the SSR regolith, the osmotic (solute) forces corresponding to the attraction between water molecules and cationic solutes^40^, and the high evaporation rates can also play prominent roles in capillary rise. The capillary rise is inversely proportional to gravity and the radius of the capillary column. The low Martian gravitational pull (0.38 of the Earth) is thus quite favourable for a higher capillary rise. This capillarity can be as high as >2.6 times that of the Earth if we consider only the differential gravity while keeping the liquid density, capillary column radius, and surface tension constant. However, the fine-textured dust in the SSR^47^ provides extremely narrow capillary columns that further enhance the capillarity.

### Conclusions and future implications

Our robust geostatistical analyses provide compelling evidence of spatial autocorrelations (for at least 60.4% of SSR at a 95% confidence limit and Moran’s I values of ≥0.93) and high concurrence rates among Cl, Fe, and hydration levels (with an adjusted R^2^ value of 60% for 95.4% of SSR at a 95% confidence level). We also report the high spatial correlation (Fig. 1e) of the SSR with the satellite-observed yearly water vapour column as a plausible indicator of the existence of a present-day regolith-atmosphere water vapour cycle in the SSR mediated by the mechanisms that cause the appearance and disappearance of the slope streak features. Our detailed analysis of the terrain preferences and flow patterns of the slope streaks further differentiates them from dry mass movements. Because of the high concentrations of Fe, Cl, and Ca, we thus consider the transient phase state changes of brines, such as hydrates of FeCl_3_ or CaCl_2_ or combinations of both, as plausible triggering mechanisms for the dynamics of these features. The presence of these chemical species can be in the bedrock or in the dust but due to the coarse-resolution data, we cannot infer their local scale regolith compositions in the SSR. However, irrespective of their presence in the bedrock or in the dust (which can be a derivative of the bedrock), the fact remains that these chemical species are there and at local scales, they have the capability to form the eutectic salts with other less prevalent species which we have discussed in the paper. The low thermal inertia, as we have explained in the paper, can be a crucial factor for the formation of slope streaks due to the diurnal thermodynamic processes, while it can also explain their extremely ephemeral nature. A recent study by Massé *et al*.^48^ efficiently demonstrates the basis for associating water activity-related Martian features with low albedo and weak spectral signatures and proves the easy possibility of deliquescence even at low temperature and moderate soil water vapour pressure. Such experiments demonstrate that the absence of water absorptions in orbiter hyperspectral signatures does not eliminate the possibility of water activity^48^.

Our work is directed more towards establishing the evidence supporting transient water activity within the slope streaks rather than arguing about the probable flow mechanisms as we understand that it will need extensive lab-based simulations. Given the extremely transient nature of the formation of slope streaks and their lithological preferences such as dusty regions, we are not proposing a fludised granular flow (or viscous flow) mechanism. However, a recent study^49^ also suggests diurnal CO_2_ freeze-thaw cycle and mass movements triggered due to subliming CO_2_ as the source of slope streak formation. One consistent aspect between Piqueux *et al*.^49^ hypothesis and our study is that both are proposing diurnal regolith-atmosphere interactions behind the slope streak formation. A visual observation of the modelled diurnal CO_2_ frost map (Fig. 5c of Piqueux *et al*.^49^) certainly suggests some level of coincidence with the SSR. However, we also observe such level of statistical coincidences for water weight % (and also for Fe and Cl weight %, thus supporting the brine hypothesis) which are observed values as opposed to the modelled CO_2_ ice values^49^. The hypothesis of subliming CO_2_ ice as an initiator of mass movement and slope streak formation is in direct contradiction with the topographical observations made for the slope streaks, the angle of repose limitations, and the surface wetting/staining mechanism that we are discussing in the paper. Nevertheless, we acknowledge the possibility of multiple mechanisms involved in slope streak formation and recommend more extensive research in this direction. Our focus is on a probable surface staining/wetting mechanism, as it can explain the largely varying sizes that slope streaks exhibit through the sudden brine formation that stains the surrounding dusty regolith through capillarity coupled with low Martian gravity which surely are prominent (but widely overlooked) factors considering the presence of dust in SSR. Once the deliquescence starts due to the hydrated salt eutectics and moisture, depending on the duration of the transient balance between the required conditions, the brine can cover a wide range of distances, elevations, and slopes taking advantage of the capillary support and high adsorption provided by the dusty arid regolith of the SSR. This capillarity can be as high as >2.6 times that of the Earth if we consider only the differential gravity. However, the fine-textured dust in the SSR provides extremely narrow capillary columns that further enhance the capillarity. In the case of the SSR regolith, the osmotic (solute) forces corresponding to the attraction between water molecules and cationic solutes^40^ can also play prominent roles in capillary rise.

We have used only Cl, Fe, and H_2_O weight % for the GWR analysis because these are the observed lithological variables. We have not included the atmospheric variables such as average water column abundance as direct input to the GWR. Throughout the paper, we argue and provide justifications that the presence of appropriate eutectic chemical species in the SSR lithology is the prime factor for slope streak formation. Of course, atmospheric variables such as RH and temperature are important but several recent simulations and studies^3, 5, 37, 41, 48^ have now proved that they should not be the main limiting factors for brine development. Thus, for the GWR we have focused on the lithological parameters only and the results of this spatial correlation analysis are encouraging.

Our results strongly suggest that brine activity lies behind slope streak formation. The widespread areal distribution of the slope streaks warrants further exploration of the plausible contemporary Martian hydrological cycle and its implications for environmental studies and planetary protection policies. The possible water activity within the slope streaks covering a large part of the equatorial latitudes is highly significant with respect to the planetary protection policies for future robotic or manned missions to prevent the biological contamination of both Earth and Mars. The planetary protection guidelines, which recently led to diversion of the Curiosity rover to prevent the contamination of a RSL site (http://www.nature.com/news/mars-contamination-fear-could-divert-curiosity-rover-1.20544?WT.mc_id=SFB_NNEWS_1508_RHBox), may consider avoiding the SSR too during future missions. Figure 3b depicts a new observation wherein both dry and wet processes, manifesting as dust avalanches and slope streaks, respectively, can co-exist on the same slopes. These slope streaks are prone to hold ephemeral brine processes on slopes and thus may also be prone to transient wet avalanches, which subsequently can accentuate the dry mass wasting due to slope instability. Such a combination of dry and wet slope processes, as is common in the Zunil crater with massive landslides (Fig. 3b and Supplementary Fig. 8), may endanger surface missions that are operated at the base of craters in the SSR. Therefore, in addition to the natural implications for planetary protection policies, this would have consequences for landing site selection procedures for the future Mars exploration program. Cl-bearing brines, particularly FeCl_3_ in the case of slope streaks, are extremely acidic and corrosive, and this requires consideration while designing future Mars landers and rovers. These results also confirm previous conclusions^3, 5, 37, 41, 48^ regarding the highly possible brine formation scenarios throughout the Martian regolith and can have profound astrobiological and habitability implications. We have analysed the presently existing observations to provide an updated evaluation of the wet versus dry mechanism for the slope streak formations. We conclude that there are enough independent observations that are rather compatible with a wet mechanism, namely a mechanisms that involves water interchange between the regolith and the atmosphere, than with a dry one. Future observations should also be compared with both the hypotheses and the traditional explanations should be revisited temporally. Although the plausible role of SSR towards sustaining any surficial life form, as we know it, is beyond the scope of this manuscript, our findings suggest that we should not eliminate the possibility of their role in providing an extended moisture support to the subsurface.

## Materials and Methods

### Global slope streak survey

Our obvious choice for this survey was the Mars Reconnaissance Orbiter (MRO) ConTeXt (CTX) imager due to its near-global coverage at a high resolution of ~6 m. However, we noticed that there were many slope streaks that were too small in dimensions (particularly width) to be detected in 6 m-resolution images. In addition, there was a possibility that within a CTX scene, only these smaller streaks were present and we could overlook them. We therefore decided to start our inventory taking sub-meter High Resolution Imaging Science Experiment (HiRISE) camera images as base data. We rendered the stamps of both of these camera images in the JMARS software (https://jmars.asu.edu). In total, 65,998 HiRISE and 60,711 CTX scenes, available through 20 September 2016, were rendered between ±60° latitudes since the temperature conditions promoting transient brine flow are not possible beyond these latitudes. Furthermore, in previous slope streak sampling surveys, never has any slope streak been reported beyond ±40° latitudes. We first surveyed all of the HiRISE scenes covering ~3% of the terrain under investigation to identify the presence of slope streaks within them. Thereafter, we surveyed CTX scenes covering the remaining ~97% of the Martian terrain between ±60° latitudes. Due to the spatial resolution limitations of the CTX imager that covered most of the terrain under investigation, we understand that there is a possibility of future addition of a few more scene centers to our inventory. However, those additions are unlikely to affect our conclusions presented within this paper. We also accept the possibility of image interpretation error leading to misidentification of some satellite scenes as SSR but we are certain that the total probable misidentified scenes will represent only a small fraction of 1% of the total scenes (~126,000) included in the study and thus will in no manner affect the geostatistical outcomes discussed in the paper. We performed a systematic survey of these remotely sensed images to derive the global scale distribution map of slope streaks. The slope streaks were recognised in the images based on their reported morphological and albedo characteristics^6–23^ within the literature. As mentioned, instead of downloading each image and interpreting them individually which would have taken a significant amount of storage space and time, we used the excellent on-the-fly image rendering capability of the JMARS software. We rendered all the CTX and HiRISE scenes between ±60° latitudes which simultaneously also solved the issue of accounting for the differing areas covered by HiRISE and CTX scenes. In the JMARS software, we have the option of turning on and off the rendered layers (HiRISE and CTX) for finding the overlap and the JMARS software also provides a feature for identifying the overlapping scene stamps among various satellite sensors. Thus, for the overlapping areas of the scenes with slope streaks, we preferred to take the scene centers of HiRISE images (which usually happen to cover very small portion of the overlapping CTX scene) and subsequently, if we observed any slope streak in the CTX scene falling outside the HiRISE coverage, we documented the scene center for that particular CTX scene to account for the non-overlapping areas. The multi-temporal scenes of the same sensor covering the same area were documented as single scene center. The need to perform this exercise was for finding the spatial extents of slope streak distribution at the global scale and thus we had to utilise optimal spatial coverage by considering any scene that had even a single slope streak visible in it. Thus, our experimentation set-up was not in need of accounting for slope streak densities in various scenes. We further obtained the statistical hot spots for these slope streaks using the Optimised Hot Spot Analysis tool in ArcGIS 10.4 software (http://desktop.arcgis.com/en/arcmap/latest/get-started/setup/arcgis-desktop-quick-start-guide.htm).

### Geostatistical analyses

We first performed the Geographically Weighted Regression (GWR)^33^ in ArcGIS 10.4 software to see the interdependence of chemical parameters, SSR and their spatial locations (latitude-longitude) (Fig. 1d). GWR explores the spatial heterogeneity and spatial non-stationarity of a regression relationship in the presence of sufficiently numerous data points (preferably several hundreds) when the structure of the process being modeled varies across the study area, as in the present case. GWR fits a regression equation to every feature in the dataset and provides a local model of the variable under investigation by incorporating the dependent and explanatory variables of features falling within the bandwidth or proximity of each target feature. The dimensions of the bandwidth are user defined based on the kernel type, bandwidth method, distance, and adjacency to neighboring features. The inherent equations and parameter selection for running this tool are well explained in the ArcGIS 10.4 software tutorial. We considered the hydration level (Fig. 1c) in the SSR to be the dependent variable as it points towards both a possible association with salts and possible briny conditions. As explanatory or independent variables, we selected Cl (Fig. 1a) and Fe (Fig. 1b) concentrations in the SSR for this analysis. The maps shown in Fig. 1a,b and c are prepared using kriging interpolation^50^ of the Gamma Ray Spectrometer (GRS) aboard the 2001 Mars Odyssey (https://grs.lpl.arizona.edu/grs-web/specials/Smoothed_rebinned_map_data) data points in ArcGIS 10.4 software and their only purpose is the visualisation of the concurrences between SSR and the chemical species. The selected kriging method was based on spherical semivariogram model^50^ with the output cell size automatically determined by the software based on the input data point spacing. The interpolated raster layers had the same projection system as the Scene Center point shape file (Mars 2000 Sphere Geographic Coordinate System (GCS) with an equidistant cylindrical projection) so that we could overlay the scene center points on them. All these interpolated maps are comparable with the ones shown in Boynton *et al*.^31^ paper based on the GRS data. We would like to maintain that these maps in Fig. 1a,b,c and e are only for visual comparison purposes as they have been used in various Mars studies as such and the scientific community is well-versed with them. The actual geostatistical analyses have been performed entirely on the vector data. Now, for all the geostatistical analyses (including GWR and Anselin Local Moran’s I spatial autocorrelation analysis), we used the same raw observed data (that has been used to prepare the maps shown in Fig. 1a,b and c) from the GRS (https://grs.lpl.arizona.edu/grs-web/specials/Smoothed_rebinned_map_data). We plotted these datasets as point layers in ArcGIS 10.4 software via “Make XY Event Layer” tool in Data Management toolbox using the given latitude-longitude and weight % information. Then we used “Near” tool in Analysis toolbox of ArcGIS 10.4 to calculate and extract the distance and additional proximity information for each point in the Scene Center point shape file layer from the corresponding closest point features in Cl, Fe, and H_2_O weight % feature classes. The attribute table of the Scene Center point shape layer thus got updated and based on the new IDs added corresponding to the weight % layers, we selected the common fields and exported corresponding weight % data from Cl, Fe, and H_2_O weight % layers to the new attribute table of Scene Center point shape layer using “Join Field” tool of Data Management toolbox. Now, the updated Scene Center point shape layer had all the information needed to perform the geostatistical analyses. Thus, we had a ready point vector layer to run the GWR tool using Scene Center point layer as input layer, H_2_O weight % as dependent variable, and Cl and Fe weight % as explanatory variables. With efficient use of several GIS operations (Make XY Event Layer, Near, and Join Field) we incorporated all the needed information (scene center positions, and corresponding chemical species’ weight %) within a single point shape file that could directly act as an input to the GWR processing. Thus, in the geostatistical analyses, we avoided the involvement of raster layers, associated interpolation offsets, and raster to vector data transfers, further maintaining the robustness of our analysis.

As an additional test for finding the possible statistically robust spatial autocorrelations within the variables mentioned above, we performed Anselin Local Moran’s I spatial autocorrelation analysis^35^ and identified the clustering events using the ArcGIS 10.4 software Cluster and Outlier Analysis tool (Fig. 2). The first step was to check whether the data were normally distributed, as any skewness could give rise to high error variance and standard errors. We used an open source geostatistical tool, GeoDa (http://geodacenter.github.io), to analyse the data distribution through Box Plots. As is evident in Fig. 2b, for the SSR, all three concentrations (Cl, Fe, and hydration levels) showed perfect normal distributions with the absence of any outlier. Next, we ran the ArcGIS 10.4 software Cluster and Outlier Analysis tool based on Anselin Local Moran’s I spatial autocorrelation^35^ to test for the spatial autocorrelation among areal units. The inherent equations and parameter selection for running this tool are well explained in the ArcGIS 10.4 software tutorial. In simple terms, the mean and the variance for the test attribute are calculated and the deviation from the mean for each feature is then multiplied by neighboring features to create a cross-product (index value), while the calculated z-scores and p-values represent the statistical significance of the computed index values. If significant numbers of neighboring features have high or low index values, then there is clustering. The z-scores and p-values are measures of statistical significance for deciding the null hypothesis of geographical randomness (no spatial clustering) in data, i.e., they indicate whether the apparent similarity (a spatial clustering of either high or low values) or dissimilarity (a spatial outlier) is more pronounced than one would expect in a random distribution. A high positive z-score indicates a cluster of features with similar values (either high or low), while a low negative z-score indicates a statistically significant spatial data outlier. In either instance, the p-value for the feature must be small enough for the cluster or outlier to be considered statistically significant. To provide statistical robustness to our analysis, we opted for a confidence level of 95% that corresponded to p-values < 0.05 and z-scores <−1.96 or >+1.96. The maps in Fig. 2a reflect this 95% confidence level. We further used GeoDa (http://geodacenter.github.io) freeware to obtain Moran Scatter Plots for the variables (Fig. 2c). The horizontal axis represents the standardised standard deviation values and is also known as the response axis. The vertical axis denotes the weighted average or spatial lag of the corresponding standardised standard deviation values on the horizontal axis. The obtained Moran’s I values in all three cases were ≥0.93 and thus indicated extremely high autocorrelations. We further checked the significance of these Moran’s I values by performing randomisation at 999 permutations. In all three cases, the pseudo p-value was 0.001 and thus significant with a normal distribution of z-values (histograms in Fig. 2c). The description of these parameters can be found in the GeoDa tutorial (http://geodacenter.github.io) and Anselin^35^.

### Climatic maps

We ran the scripts for version 5.2 of the Mars Climate Database (MCD)^44, 45^ on the Interactive Data Language (IDL) platform to generate the annual maps of climatic parameters presented in Supplementary Figs 2b, 3a, 4 and 6.

### Georeferencing of physio-chemical and climatic maps

To observe the global slope streak distribution with respect to various physio-chemical and climatic parameters, we georeferenced all the maps derived from the past studies and assigned them the same projection as the SSR points, i.e., the Mars 2000 Sphere Geographic Coordinate System (GCS) with an equidistant cylindrical projection using ArcGIS 10.4 software.

### Morphometric analysis

We first mapped ~1000 slope streak samples using five high-resolution HiRISE DTMs and corresponding orthorectified images (Supplementary Fig. 1b). The reported accuracy of these DTMs by the HiRISE scientific team is in centimeter scales. The slope streak samples represented different latitudes and SSR: Bullseye Crater (DTM: DTEEC_003543_1910_003398_1910_A01), a crater in Elysium Planitia (DTM: DTEEC_004151_1810_020963_1810_U01), Tooting Crater (DTM: DTEEC_005771_2035_003569_2035_U01), a crater in Arabia Terra (DTM: DTEEC_008520_2085_009232_2085_A01), and lava marks in Kasei Valles (DTM: DTEED_033617_1990_034316_1990_A01).

First, we maintain that our methodology of terrain analysis is quite different from the one adopted by Brusnikin *et al*.^21^. We have relied on the DTMs prepared by the HiRISE team with best possible accuracy, while Brusnikin *et al*.^21^ have used PHOTOMOD software for deriving the terrain information from the stereopairs through image matching without creating the DTMs. Brusnikin *et al*.^21^ have reported a probable ~0.5 m vertical precision for full-resolution images providing slope measurement accuracy of ~2° for short ~20 m segments. On the other hand, the HiRISE DTMs provided by the HiRISE team are although very few in numbers but are extremely reliable with the reported vertical precision of a few tenths of a meter, indicating a stereo matching precision approaching 0.2 of a pixel (Kirk *et al*.)^51^. Thus, we are not arguing against the methodology used by Brusnikin *et al*.^21^ as we understand that due to the different approaches adopted and variations in the precision of measurements, the results of Brusnikin *et al*.^21^ study can be slightly varying with our results. Brusnikin *et al*.^21^ surveyed 19 stereo pairs with over 700 streak samples. As there are very few DTMs provided by the HiRISE team that abundantly contain slope streaks, we carefully selected five such DTMs well distributed across the Martian terrain (Supplementary Fig. 1b) with considerable number of slope streaks. We accounted for the lesser number of DTMs (5 against 19 stereopairs of Brusnikin *et al*.^21^) by mapping ~1000 slope streak samples (~700 by Brusnikin *et al*.^21^).

Now, coming to the inherent noises within the HiRISE DTMs, we certainly avoided all of them up to a considerable extent. There are four such artifacts as reported by the HiRISE team (http://www.uahirise.org/dtm/about.php) but a notable point here is that these noises are present only in several DTMs: (1) boxes, (2) CCD seams, (3) faceted areas, and (4) manually interpolated areas. All of these artifacts are very clearly identifiable in the terrain shaded relief (examples given on http://www.uahirise.org/dtm/about.php). While selecting the five DTMs for our analyses, finding such artifacts using terrain shaded relief views was an additional criterion. We made sure that in none of the selected DTMs, such artifacts were present in the slope streak regions. In case of several slope streaks which were adjoining the regions of probable artifacts, we preferred to leave them out from the sampling and analyses.

The abrupt fluctuations in the slope as seen in profiles of Fig. 4 are understandable considering the extremely high resolutions of the used DTMs and the highly undulating Martian terrain. One thing to notice here is that the abruptness in the slope values given in the profiles are due to the fact that they are not representing the average values at different elevations for an entire slope streak but they are absolute slope values for each pixel along only a transect within the streak. The averaged-out slope values for an entire streak are certainly moderate and not so much fluctuating. However, here we needed the profiles along transects to discuss our hypotheses. We used Slope, Aspect, and Curvature tools given in the Spatial Analyst toolbox of ArcGIS 10.4 software to derive the respective morphometric layers. For each cell, the Slope tool calculates the maximum rate of change in value from that cell to its eight neighbors^50^, while the Aspect tool calculates the direction of that slope, i.e., the downslope direction of the maximum rate of change in value from each cell to its eight neighbors^50^. The Curvature tool calculates the second derivative value of the input surface on a cell-by-cell basis as for each cell, a fourth-order polynomial is fit to a surface composed of a 3 × 3 window^52, 53^. The surface roughness was derived using the Geospatial Data Abstraction Library (GDAL) roughness tool within QGIS 2.16 software. Roughness tool calculates the largest inter-cell difference of a central pixel and its surrounding cells^54^ for each cell.

Now, coming to the calculation of the average terrain preference, we first digitised and mapped all the slope streak samples using orthorectified images corresponding to the DTMs. We classified the morphometric layers in various classes as shown in Fig. 4d. Next, we clipped all these layers using the polygon shape file of the mapped samples and for each morphometric class, we derived the percent of total pixels representing that particular class. For calculating the average trigger point preference, we first marked the topmost points (trigger points) of all the sampled slope streaks as separate point shape file and then used “Extract Values to Points” tool from Spatial Analyst toolbox of ArcGIS 10.4 to extract corresponding morphometric information to the attribute table of the trigger points. Finally, we calculated the percent of total points within separate morphometric classes.

### Slope streak transect analysis

The transect analysis was performed in ArcGIS 10.4 software. The elevation and slope values for the pixels coinciding with the drawn transects were extracted using the “Add Surface Information” tool in the ArcGIS 10.4 software.

### Rate of appearance calculation

For comparison purposes, we used the same formula as suggested by Aharonson *et al*.^19^ to calculate the % rate of appearance on the repeat HiRISE images of two different craters in the Elysium Planitia region. The image pairs were: (1) PSP_002252_1880_RED/ESP_046360_1880_RED, and (2) PSP_004151_1810_RED_A_01_ORTHO /ESP_020963_1810_RED_A_01_ORTHO. Although we have performed this appearance rate analysis on only two image pairs, our main aim was to lengthen the temporal scale from several years to nearly a decade, which has never been reported before for HiRISE images. We have achieved this objective of long-term monitoring through these stereopairs and we acknowledge that this appearance rate may vary at local scales among the SSR.

### Data availability

The datasets used for geostatistical analysis during the current study are available in the GRS repository (https://grs.lpl.arizona.edu/grs-web/specials/Smoothed_rebinned_map_data). All data generated or analysed during this study are included as references in this published article (and its Supplementary information file).

### Ethical approval and informed consent

We confirm that this is a completely remote sensing-based interplanetary study and does not involve any biological experiments.

## Electronic supplementary material

Supplementary Information
