# Supplementary material for: Martian slope streaks as plausible indicators of transient water activity
Source: Sci Rep. 2017 Aug 1;7:7074. doi: 10.1038/s41598-017-07453-9 (PMC5539097; doi:10.1038/s41598-017-07453-9)
Supplement: Supplementary file 1 — Supplementary Information [file 41598_2017_7453_MOESM1_ESM.doc]

**Martian slope streaks as plausible indicators of transient water activity**

Anshuman Bhardwaj^1*^, Lydia Sam^2,3^, F. Javier Martín-Torres^1,4^, María-Paz Zorzano^1,5^, Ricardo M. Fonseca^1^

^1^Division of Space Technology, Department of Computer Science, Electrical and Space Engineering, Luleå University of Technology, Luleå, Sweden

^2^Institut für Kartographie, Technische Universität Dresden, Germany

^3^Department of Environmental Science, Sharda University, Greater Noida, India

^4^Instituto Andaluz de Ciencias de la Tierra (CSIC-UGR), Armilla, Granada, Spain

^5^Centro de Astrobiología (INTA-CSIC), 28850 Torrejón de Ardoz, Madrid, Spain

^*^Corresponding Author

**Supplementary information**

In this section, we mention several additional considerations in favor of possible wet mechanisms for slope streaks on Mars. Although we need extensive research to establish these considerations as definite markers of wet mechanisms, we mention them here in an attempt to elucidate the propensity of SSR for ephemeral wet scenarios. Similar to the high concentrations of iron oxides, the Cl enrichment of Martian regolith is also widely attributed to past water cycles controlling the processes of water transport, hydrothermal alteration, and evaporation^30,40^. The numerical simulations for the global hydrology of early Mars^S1^ further confirm that all of the SSR have always been hydrologically dynamic with significant groundwater upwelling and evaporation depicting an active water cycle (Supplementary Fig. 2d). We understand that these simulations for 500 Myr of the pre-Tharsis era display completely different climatic and surficial hydrological settings from the present-day Mars. However, the crustal similarities, lithology, and bed rock geology are a few of the subsurface parameters that possibly can display similar types of capillarity even now as they displayed in the past for the significant ground water upwelling. The plausible connection with a sustained atmosphere-regolith water cycle is shown by the positive correlation of the SSR with the regions of shallowest depth to the water table (<1 km) and the highest cumulative past evaporative groundwater loss (2.5-5 km), which also points towards the extremely favorable regolith conditions for capillary rise^S1^. The coexistence of slope streak scene centers with anomalous (both positive and negative) gravity regions (96%) (Supplementary Fig. 2c) and low-to-medium crustal thickness (89% for 10-60 km) (Supplementary Fig. 1d) is evident in the recently released gravity maps^S2^. Although this coexistence demands a separate global-scale geological analysis, the immediate inferences suggest differential bedrock compositions, buried water channels, groundwater reservoirs, or subsurface ice, further signifying a hydrologically active regime in the SSR.

The southernmost slope streaks observed in the sandy terrain of Russell Crater (Supplementary Fig. 7) at ~55°S present an interesting picture as they could be observed on the same sandy slopes as the CO_2_ sublimation gullies and Dark Dune Spots (DDS)^S3,S4^ (Supplementary Fig. 7b). The morphological differences between the sublimated arid slope streaks and the DDS signify the involvement of H_2_O in the former case. The DDS have been reported as seasonal manifestations^S3^ unlike the slope streaks as they start to appear during late winter and spring on the ice cover over dark sand sheets within a crater. Sand dunes display unique thermodynamic properties as although they are the first surfaces showing start of frosting in fall and defrosting in late winter or early spring, frost persists on dunes longer than the surrounding terrains^S5^. Malin and Edgett (2000)^S5^ attribute this uniqueness not only to the thermophysical properties of sand but also to the trapping and release of volatiles (CO_2_ and H_2_O) within sand deposits through sublimation. Malin and Edgett (2000)^S5^ further suggest CO_2_ to be the frost that disappears in early spring showing the initial phase of sand defrosting (consistent with the season of appearance of DDS^S3^), and H_2_O to be the late sublimation entity prolonging the dune defrosting well into the late summer. This delay in the sublimation does not only suggest the possibility of water activity in the slope streaks but can also play a major role in explaining the significantly different morphological characteristics of slope streaks and DDS.

Whether the water vapour is sufficient to create slope streaks which are hundreds to thousands of meters long is a different question altogether and first needs answering what the ratio of salt-to-soil should be to change the viscosity of SSR soils and fluidise it. This is beyond the scope of the present work as answering it would need more observations and rigorous simulations for identifying salts at local scales and compare their phase diagrams. The only *in-situ* surface measurements of the full diurnal and seasonal water cycles so far have been provided by the Rover Environmental Monitoring Station (REMS). Comparisons with other Curiosity rover instrument Sample Analysis at Mars (SAM) show a day-to-night variability in the near-surface water content at Gale Crater of about 4-6 pr-µm that seems reasonable^S6^. All the calculations of atmospheric water assume that water vapour is uniformly mixed in the vertical height within the boundary layer, but this is certainly not the case if there is a diurnal interchange of water. REMS has been measuring for the last 2 Martian years the diurnal and seasonal water cycles at the equator, and has observed nigh-time maxima between 2-4 H_2_O pr-µm^S6^. The noticeable point here is that the yearly average of atmospheric water vapour column observations are about one order of magnitude greater (Fig 1d) and that the Mars Global Surveyor Thermal Emission Spectrometer (TES) day-time climatology shows values of about 10-14 pr-µm at the site of REMS^S6^. Thus, there is a great uncertainty on the total amount of available atmospheric water and it points towards variability between day and night. During the day, the ground would become a source of water vapour due to desorption whereas during the night the ground would become a sink^S6^. Wet mechanisms for both RSL and SSR share a common explanatory issue: the challenge of identifying sufficiently large H_2_O source(s). Subsurface ice or aquifers remain undetected at RSL sites, and the volume of brine production via deliquescence remains unquantified. However, according to our geostatistical analyses, subsurface water is present for the SSR, which is mostly distributed in the northern hemisphere. RSL are mostly distributed in the southern hemisphere, where observations support chemical association of H_2_O with S^S7^ including the possibility of Iron sulfates as a key mineral group. The mechanism for formation of RSL and SSR may both involve water interchange between regolith and atmosphere, however supported by different chemical substrates, and thus implying different temperature/RH conditions, seasonality (for RSL), and formation rates.


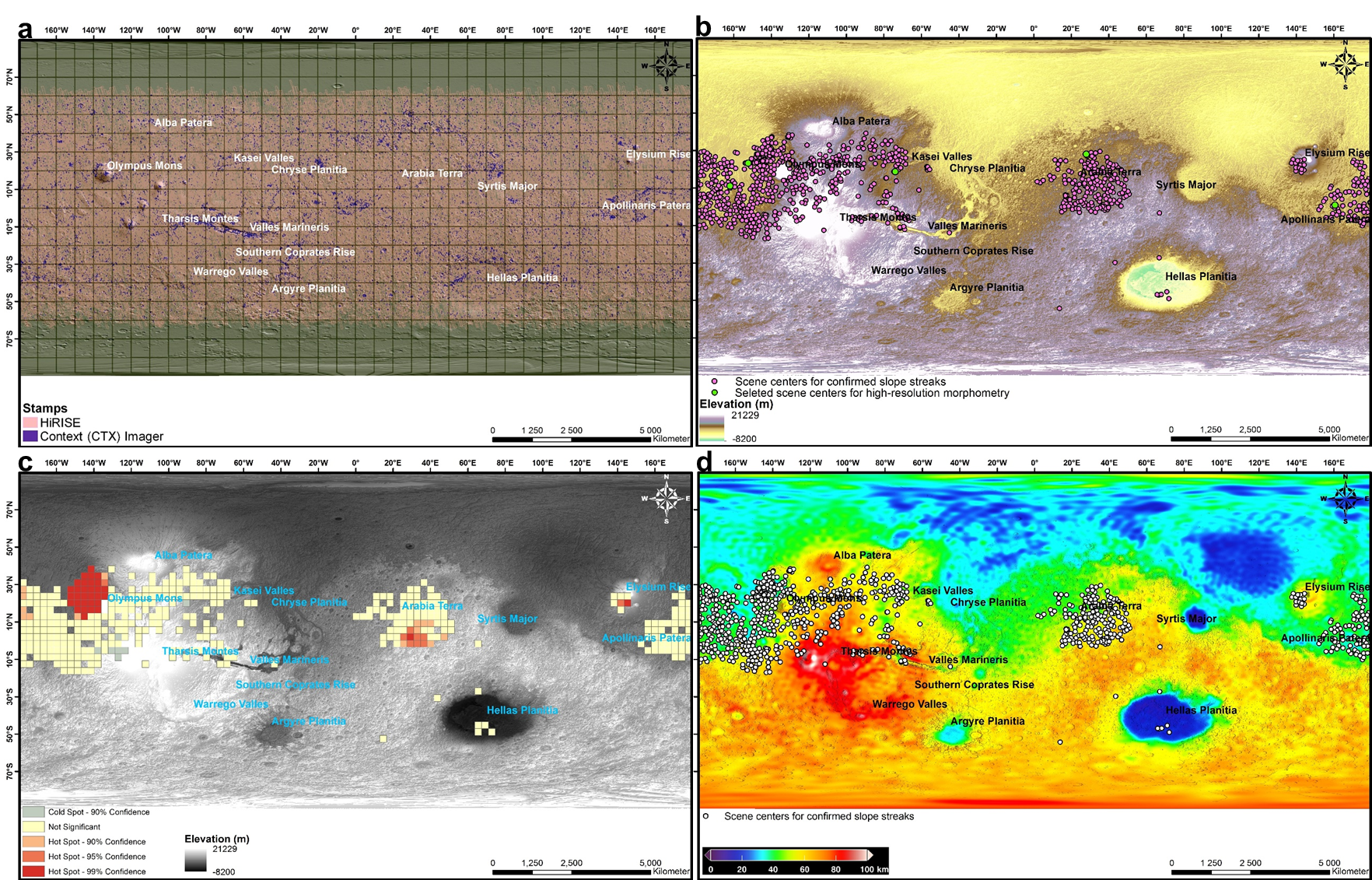


**Supplementary Figure 1** | **Surveyed images and slope streak hot spots. a,** > 95% global coverage of the surveyed High Resolution Imaging Science Experiment (HiRISE) and Context (CTX) imager scenes. **b and** **d,** Scene centers of the confirmed slope streak images with Mars Orbiter Laser Altimeter (MOLA) elevation and crustal thickness^S2^, respectively, in the background. **c,** Hot spots of slope streaks on Mars based on Optimized Hot Spot Analysis in ArcGIS 10.4^28,29^: (1) west of Olympus Mons, (2) southern part of Arabia Terra, and (3) south of Elysium Rise. The Maps are created using ArcGIS Version 10.4 (http://desktop.arcgis.com/en/arcmap/latest/get-started/setup/arcgis-desktop-quick-start-guide.htm). HiRISE image credit: NASA/JPL/University of Arizona. CTX image credit: NASA/JPL-Caltech.


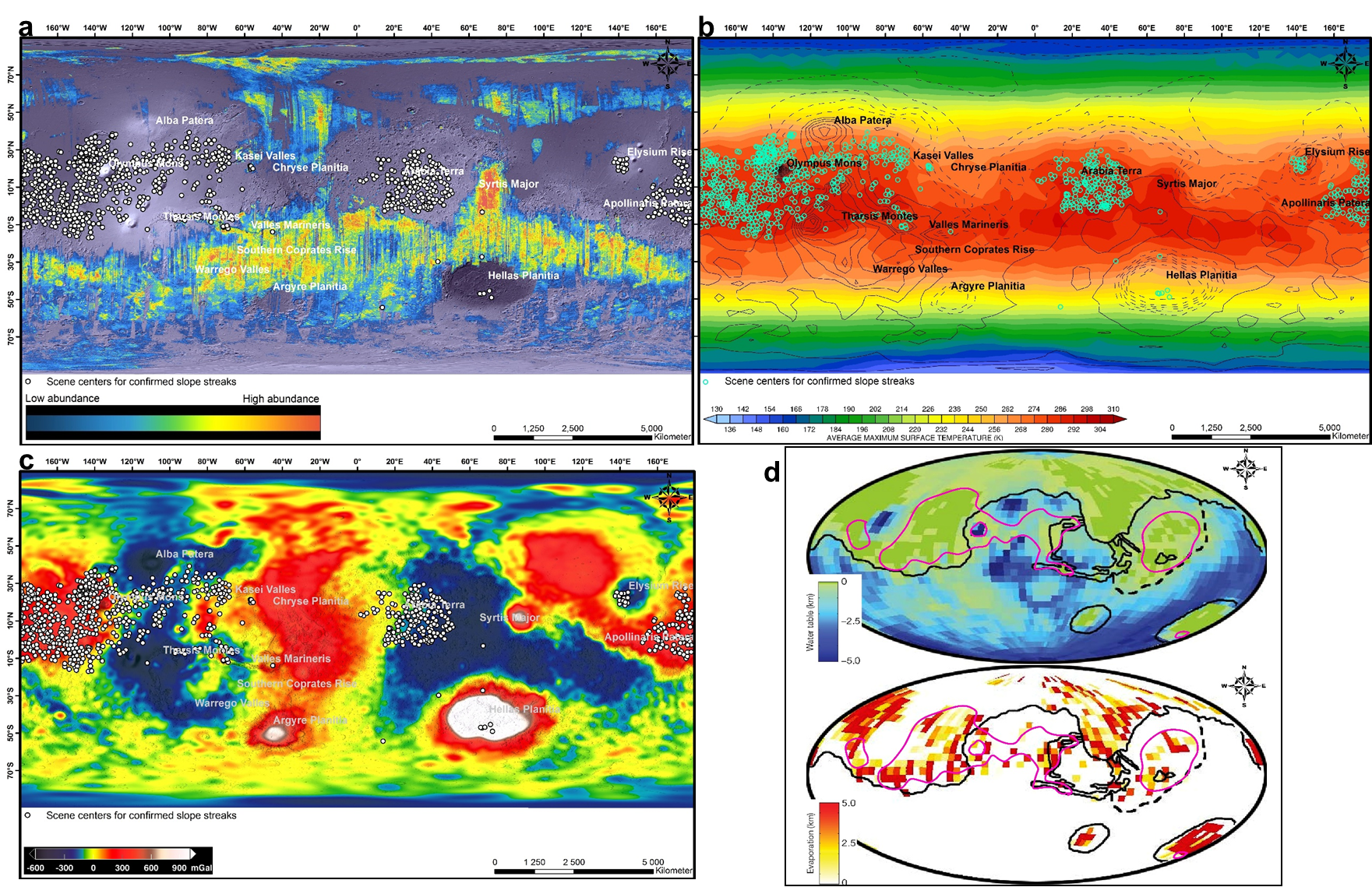


**Supplementary Figure 2** | **Observed slope streaks and several estimated and modeled parameters. a,** Pyroxene abundance^32^. **b,** Average annual maximum surface temperature^44,45^. The dashed and solid black contours represent negative and positive elevations, respectively, at 1000 m intervals. **c,** Global Bouguer gravity anomaly^S2^. **d,** Modeled depth to the water table (top) and cumulative evaporative groundwater loss (bottom)^S1^. The pink boundary encloses the regions of slope streaks. The Maps are created using ArcGIS Version 10.4 (http://desktop.arcgis.com/en/arcmap/latest/get-started/setup/arcgis-desktop-quick-start-guide.htm).


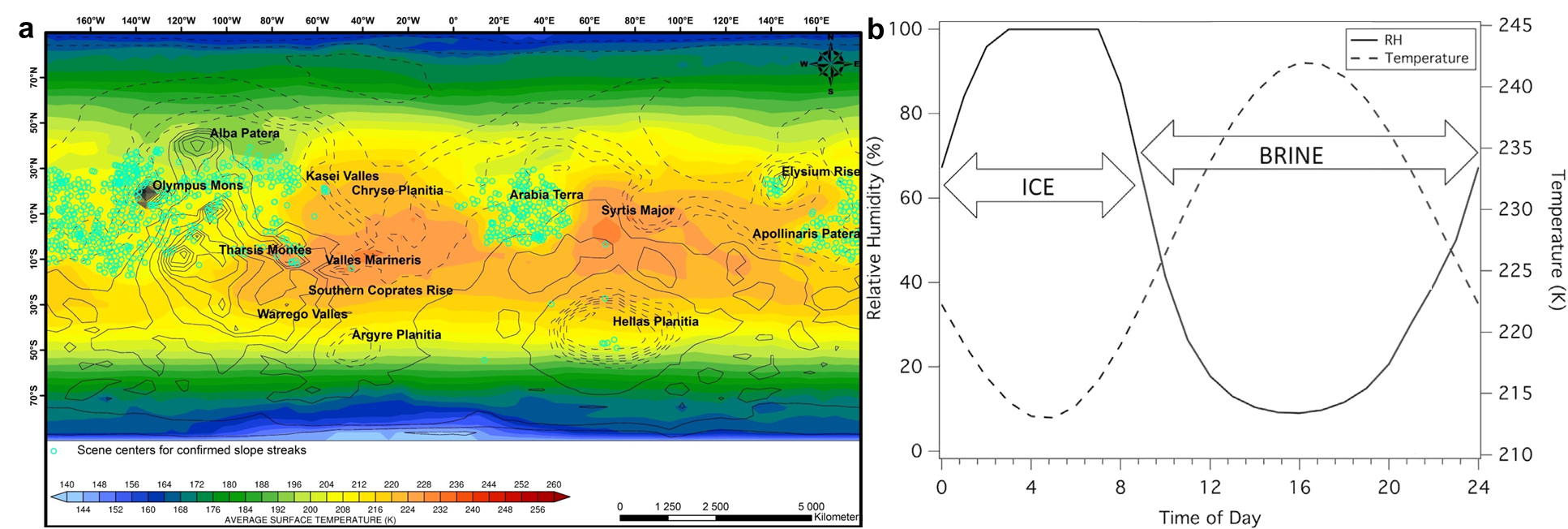


**Supplementary Figure 3 | Yearly average surface temperature on Mars and its implications for CaCl_2_ brines.** **a,** Yearly average surface temperature on Mars^44,45^. The dashed and solid black contours represent negative and positive elevations, respectively, at 1000 m intervals. **b,** Diurnal phases of the CaCl_2_/H_2_O system depending on the temperature and relative humidity conditions within a surface skin^41^. The Map is created using ArcGIS Version 10.4 (http://desktop.arcgis.com/en/arcmap/latest/get-started/setup/arcgis-desktop-quick-start-guide.htm).


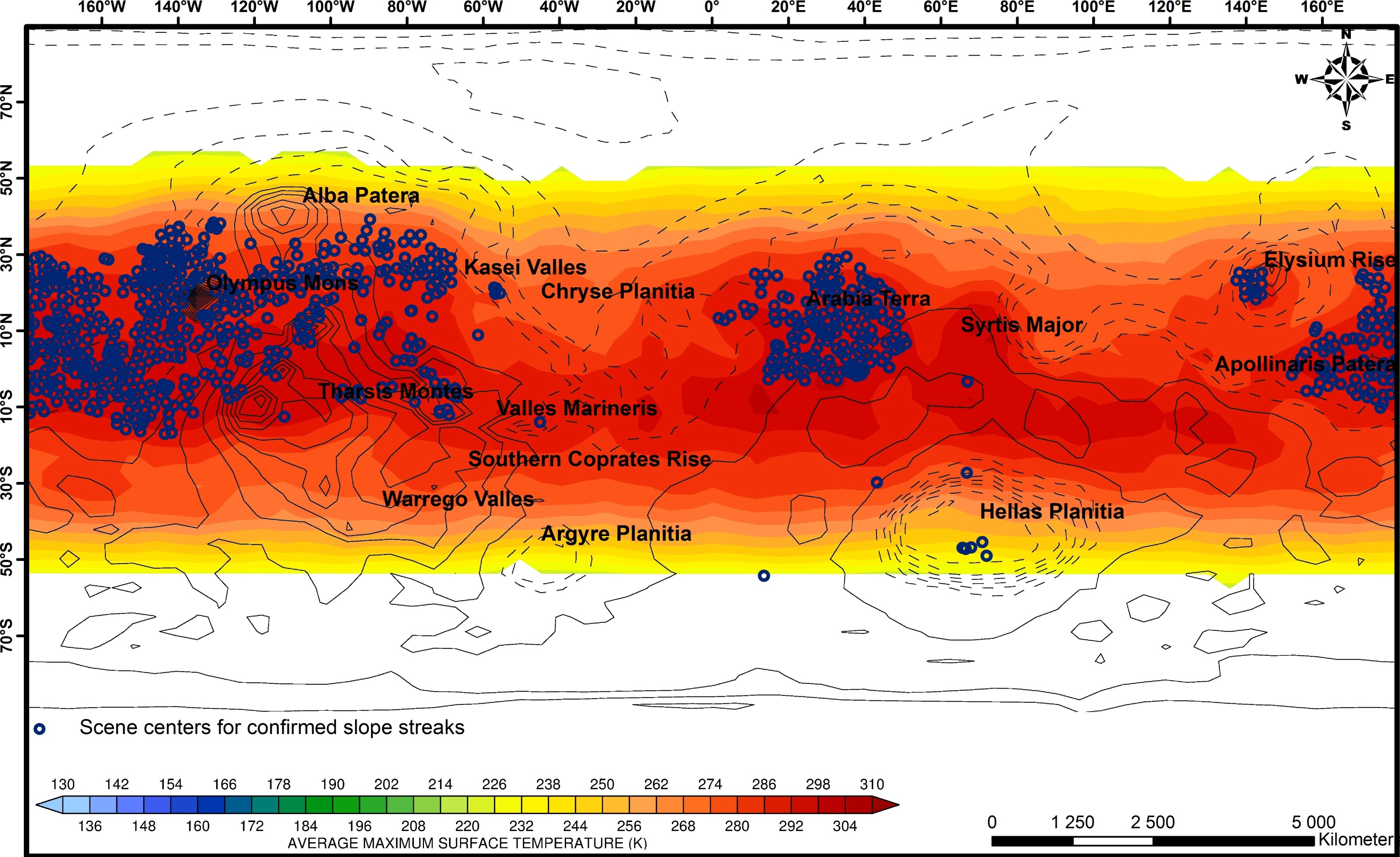


**Supplementary Figure 4 | >223 K average maximum surface temperature^44,45^.** All of the SSR fall within this temperature range. The dashed and solid black contours represent negative and positive elevations, respectively at 1000 m intervals. The Map is created using ArcGIS Version 10.4 (http://desktop.arcgis.com/en/arcmap/latest/get-started/setup/arcgis-desktop-quick-start-guide.htm).


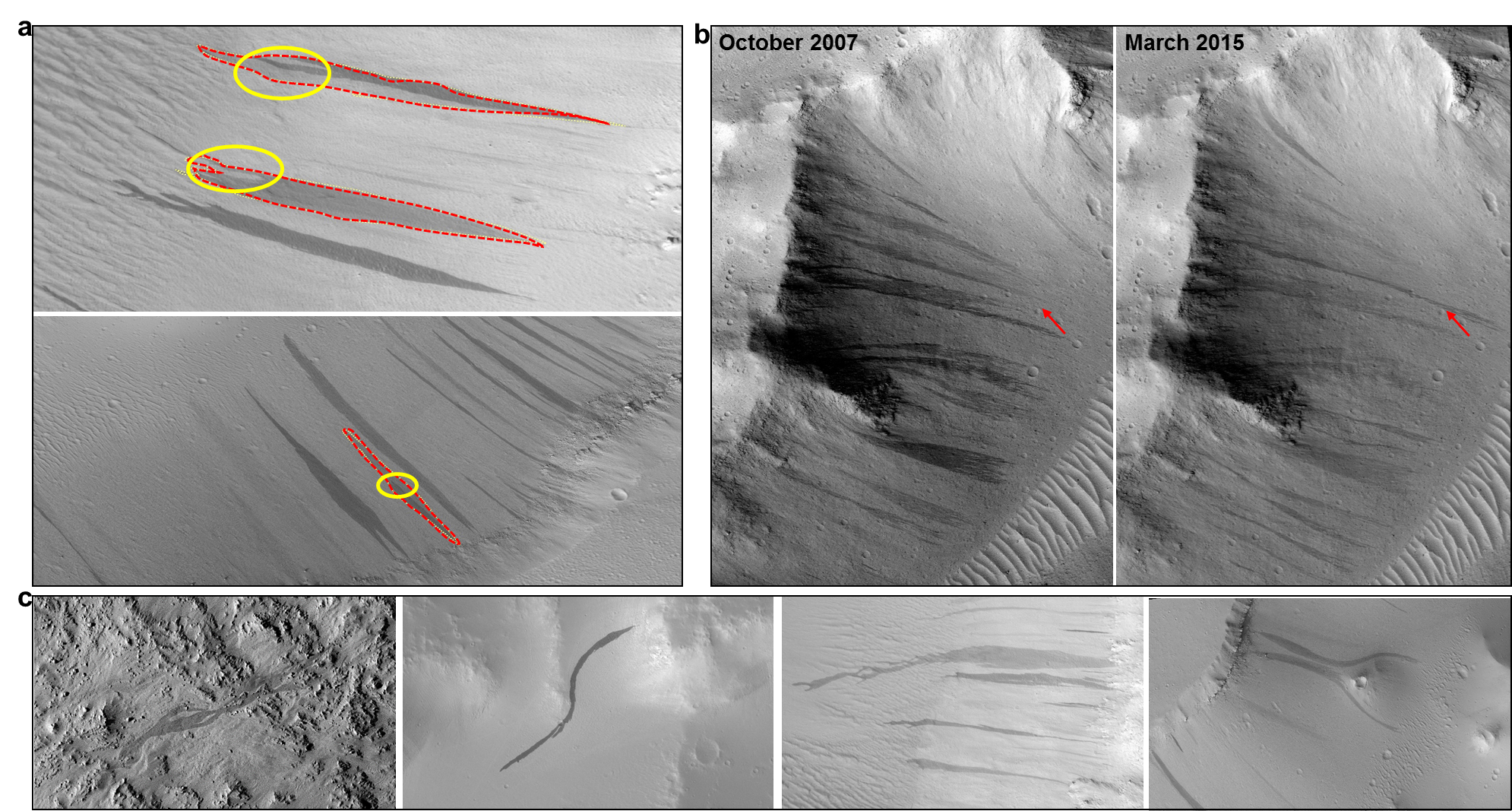


**Supplementary Figure 5** | **Regeneration, elongation, and flow patterns of the slope streaks in HiRISE images. a,** Regeneration (darker albedo) within the previous streak boundary (dashed red polygons). Careful observation within the yellow ellipses can show the differential albedo of the previous and the newer slope streaks. **b,** The red arrow shows the rare elongation of a slope streak. **c,** Flow patterns suggestive of wet mechanisms. HiRISE image credit: NASA/JPL/University of Arizona


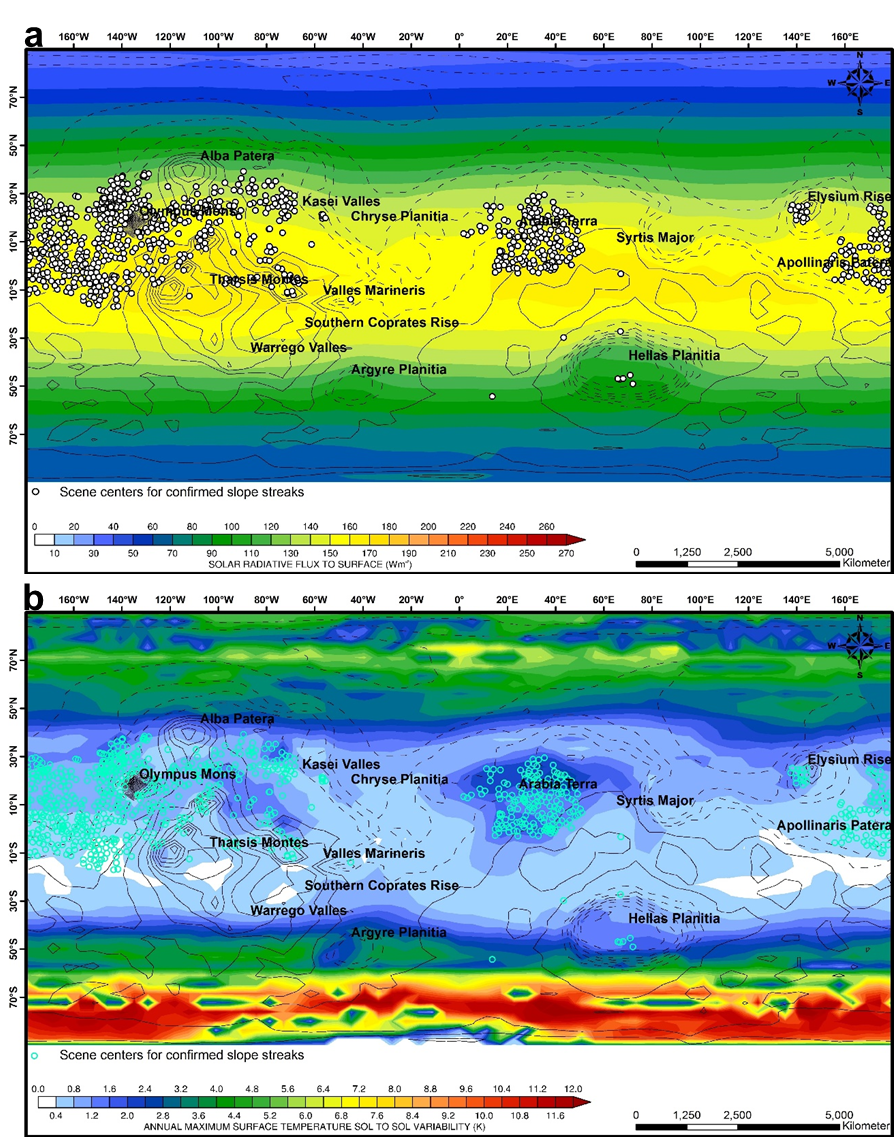


**Supplementary Figure 6** | **Modeled climatic parameters^44,45^. a,** Average annual solar radiative flux to surface. **b,** Sol-to-sol variability in annual maximum surface temperature (lower values in slope streak regions support a lack of seasonality). In both maps, the dashed and solid black contours represent negative and positive elevations, respectively, at 1000 m intervals. The Maps are created using ArcGIS Version 10.4 (http://desktop.arcgis.com/en/arcmap/latest/get-started/setup/arcgis-desktop-quick-start-guide.htm).


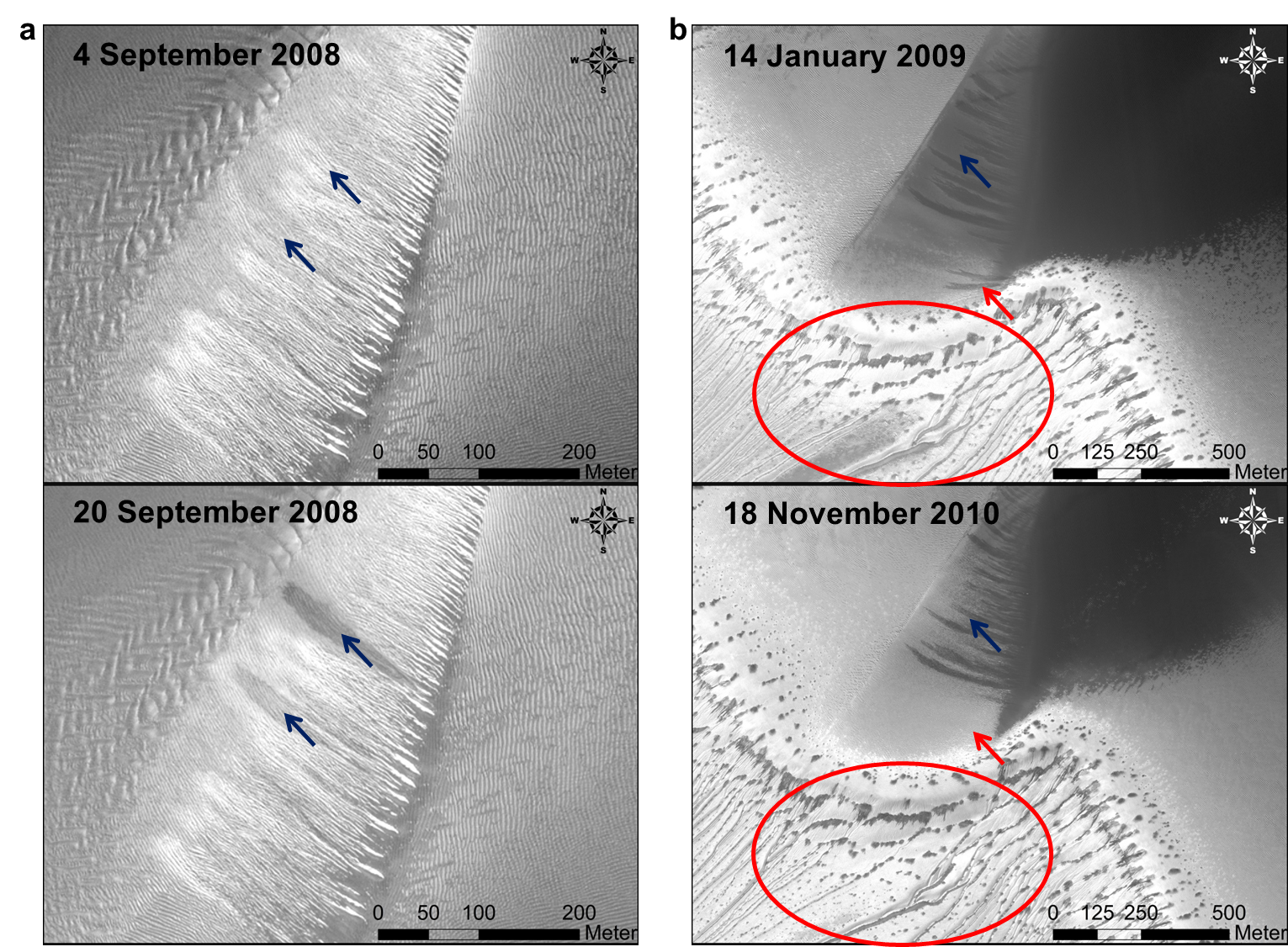


**Supplementary Figure 7 | Southernmost slope streaks. a,** Emergence of new slope streaks (blue arrows). **b,** Appearance (blue arrows) and disappearance of slope streaks (red arrows). The different morphology of the slope streaks from the CO_2_ sublimation gullies (red ellipses) is evident. The Maps are created using ArcGIS Version 10.4 (http://desktop.arcgis.com/en/arcmap/latest/get-started/setup/arcgis-desktop-quick-start-guide.htm). HiRISE image credit: NASA/JPL/University of Arizona


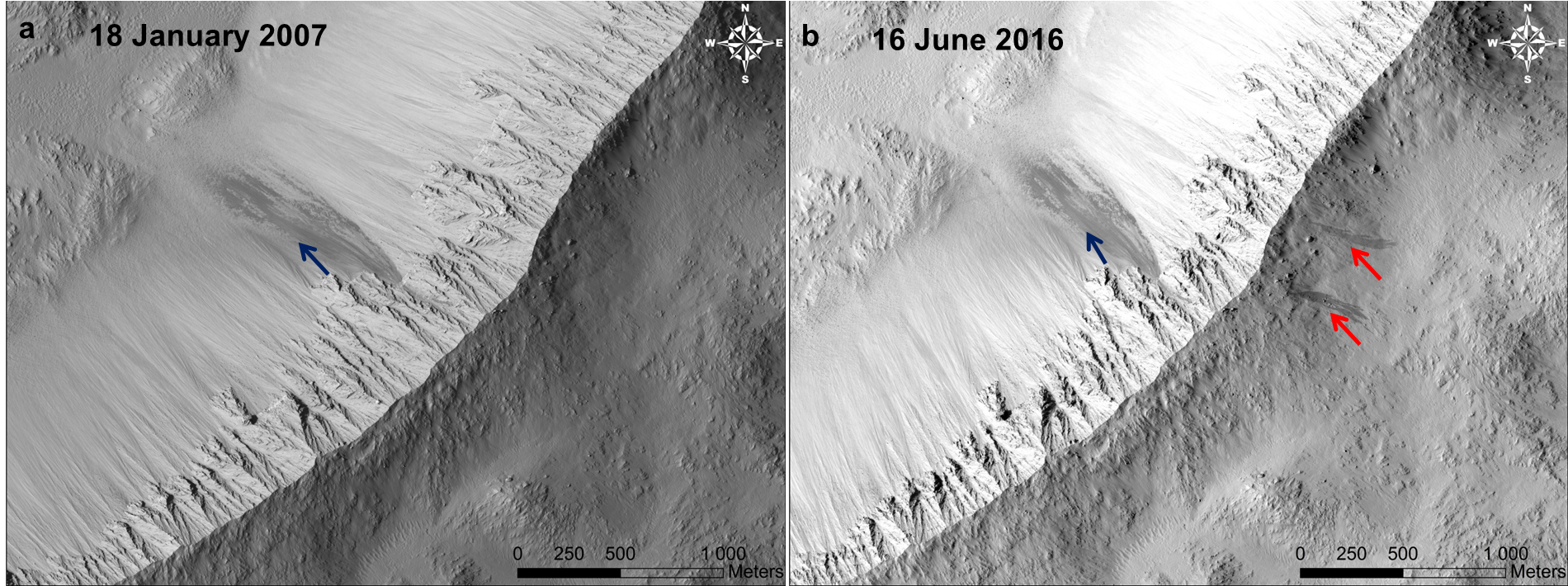


**Supplementary Figure 8 | Dry and wet slope processes in Zunil crater. a,** Blue arrow shows a massive dry mass movement on the north-west-facing slopes of Zunil crater. **b,** Red arrows show newly formed slope streaks at the rim of Zunil crater. The Maps are created using ArcGIS Version 10.4 (http://desktop.arcgis.com/en/arcmap/latest/get-started/setup/arcgis-desktop-quick-start-guide.htm). HiRISE image credit: NASA/JPL/University of Arizona

**
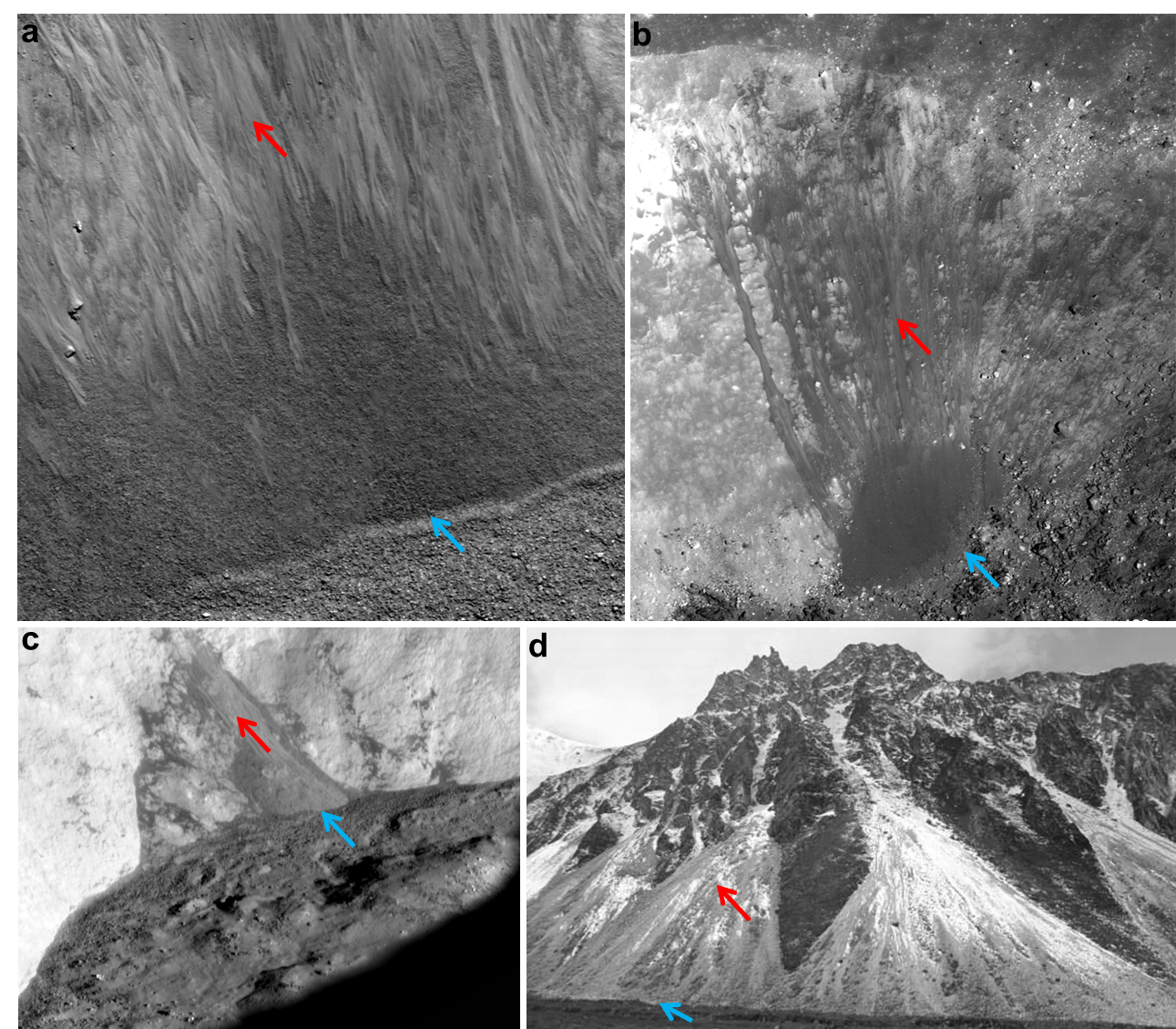
**

**Supplementary Figure 9 | Lunar (a, b, c) and terrestrial (d) analogues for the dry mass wasting shown in Fig. 3. Lunar observations are made using Lunar Reconnaissance Orbiter Camera (LROC) Narrow Angle Cameras (NACs)^S8-S10^ courtesy of NASA/GSFC/Arizona State University. The red arrows in all the figures show the dry talus slopes while the blue arrows show the talus deposits. a,** Dry flows on the slopes of Furnerius A crater wall (Scene ID: NAC M187848982RC) (http://wms.lroc.asu.edu/lroc/view_lroc/LRO-L-LROC-3-CDR-V1.0/M187848982RC). Resemblance with Fig. 3b is striking. **b,** Dry debris flows along the wall of Schubert A crater (Scene ID: NAC M141743432LE) (http://wms.lroc.asu.edu/lroc/view_lroc/LRO-L-LROC-2-EDR-V1.0/M141743432LE). **c,** Dry granular debris flow on the wall of Dugan J (Scene ID: NAC M1131216329RC) (http://wms.lroc.asu.edu/lroc/view_lroc/LRO-L-LROC-3-CDR-V1.0/M1131216329RC). **d,** Dry mass movements (scree falls) in Langtang Valley, Nepal Himalaya. The picture has been taken by the first author in May 2014. The images shown in Fig. a, b, and c have been exported using ArcGIS Version 10.4 (http://desktop.arcgis.com/en/arcmap/latest/get-started/setup/arcgis-desktop-quick-start-guide.htm). All the LROC images have been downloaded from the LROC Data Node of the Planetary Data System (PDS) archive (http://wms.lroc.asu.edu/lroc/search).


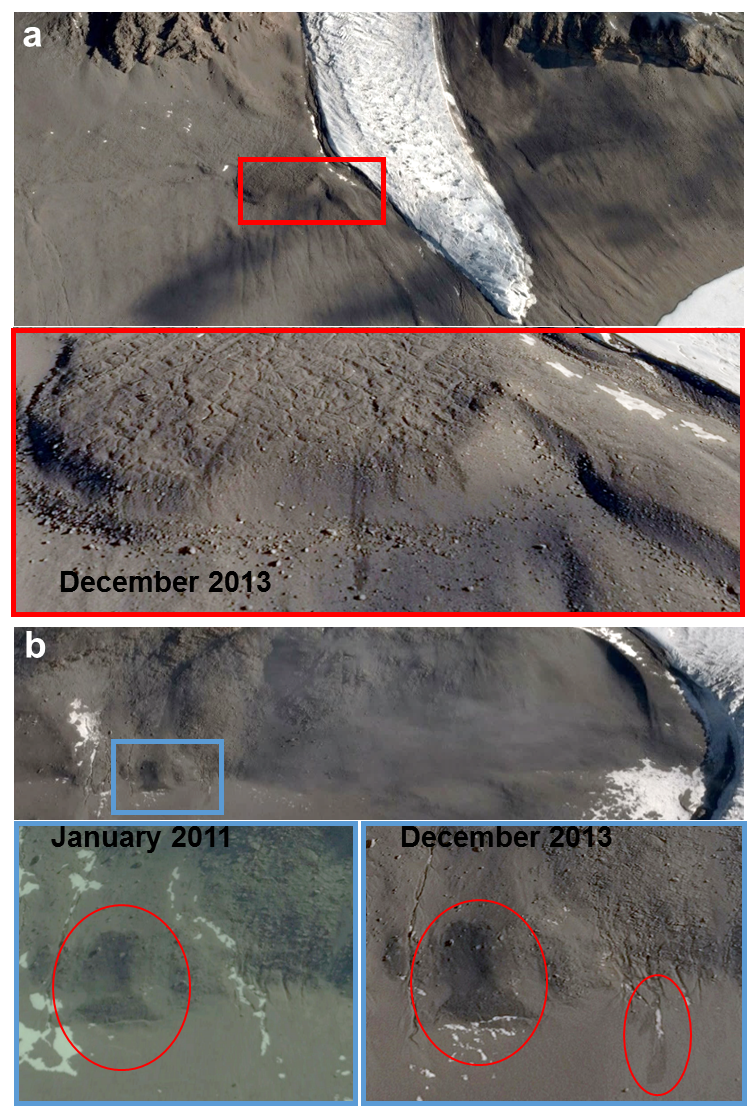


**Supplementary Figure 10 | Terrestrial analogues in Antarctic for slope streaks. a,** Beside Rhone Glacier terminus (taken from Google Earth). **b,** Wet streaks in Taylor Glacier Valley as observed in Google Earth images. The red ellipses are marking the wet streaks on the slopes. The data provider for the used Google Earth images is DigitalGlobe.

**References**

S1. Andrews-Hanna, J. C., Phillips, R. J., & Zuber, M. T. Meridiani Planum and the global hydrology of Mars. *Nature* **446(7132)**, 163-166 (2007).

S2. Genova, A. *et al.* Seasonal and static gravity field of Mars from MGS, Mars Odyssey and MRO radio science. *Icarus* **272**, 228-245 (2016).

S3. Horváth, A. T. *et al*. Morphological analysis of the Dark Dune Spots on Mars: New aspects in biological interpretation. In *33rd Lunar Planet. Sci. Conf.* **33** (Lunar and Planetary Institute, 2002).

S4. Kereszturi, A. A. *et al*. Possible liquid-like water produced seepage features on Mars. In *40th Lunar Planet. Sci. Conf.* **40** (Lunar and Planetary Institute, 2009).

S5. Malin, M. C., & Edgett, K. S. Frosting and defrosting of Martian polar dunes." In *31st Lunar Planet. Sci. Conf.* **31** (Lunar and Planetary Institute, 2000).

S6. Martínez, G. M. *et al*. Likely frost events at Gale crater: Analysis from MSL/REMS measurements. *Icarus* **280**, 93-102 (2016).

S7. Karunatillake, S. *et al.*. Sulfates hydrating bulk soil in the martian low and middle latitudes. *Geophysical Research Letters* **41(22)**, 7987-7996 (2014).

S8. Humm, D. C. *et al*. Flight Calibration of the LROC Narrow Angle Camera. *Space Science Reviews* **200(1-4)**, 431-473 (2016).

S9. Mahanti, P. *et al*. Inflight Calibration of the Lunar Reconnaissance Orbiter Camera Wide Angle Camera. *Space Science Reviews* **200(1-4)**, 393-430 (2016).

S10. Robinson, M. S. *et. al*. Lunar Reconnaissance Orbiter Camera (LROC) Instrument Overview. *Space Science Reviews* **150(1)**, 81-124 (2010).
